# Generating Patient-Derived HCC Cell Lines Suitable for Predictive In Vitro and In Vivo Drug Screening by Orthotopic Transplantation

**DOI:** 10.3390/cells13010082

**Published:** 2023-12-30

**Authors:** Lisa Staffeldt, Gregor Mattert, Kristoffer Riecken, Götz Rövenstrunk, Anika Volkmar, Asmus Heumann, Mohamed Moustafa, Manfred Jücker, Boris Fehse, Udo Schumacher, Stefan Lüth, Janine Kah

**Affiliations:** 1 Institute of Anatomy and Experimental Morphology, University Medical Center Hamburg-Eppendorf, 20246 Hamburg, Germanyuschumacher@uke.de (U.S.); 2 Brandenburg Medical School, Center for Translational Medicine, 14770 Brandenburg an der Havel, Germany; gregor.mattert@mhb-fontane.de (G.M.); goetz.roevenstrunk@mhb-fontane.de (G.R.);; 3 Research Department Cell and Gene Therapy, University Medical Center Hamburg-Eppendorf, 20246 Hamburg, Germany; 4 Department of General, Visceral and Thoracic Surgery, University Medical Center Hamburg-Eppendorf, 20246 Hamburg, Germany; 5 Department of Visceral Transplantation, University Medical Center Hamburg-Eppendorf, 20246 Hamburg, Germany; 6 Center for Experimental Medicine, Institute of Biochemistry and Signal Transduction, University Medical Center Hamburg-Eppendorf, 20246 Hamburg, Germany; juecker@uke.de; 7 German Center for Infection Research, Hamburg-Lübeck-Borstel Partner Site, 38124 Braunschweig, Germany; 8 Medical School Berlin, Mecklenburgische Straße 57, 14197 Berlin, Germany; 9 Department of Gastroenterology, University Hospital Brandenburg, 14770 Brandenburg an der Havel, Germany

**Keywords:** patient-derived HCC cell lines, in vitro investigating, therapeutic testing, orthotopic transplantation

## Abstract

Hepatocellular carcinoma (HCC) results in high mortality due to ineffective systemic therapy. Human immortalized cell lines are commonly used to study anti-tumor effects in the context of new anti-tumor therapies and tumor biology. As immortalized cell lines have limited biological relevance and heterogeneity compared to primary cells, patient-derived tumor tissues, and corresponding immune cells are the gold standards for studying the complexity of individual tumor entities. However, culturing primary HCC cells has a low success rate. Here, we aimed to establish a reproducible approach to preserve the patient-derived liver cancer cells for in vitro and in vivo studies. The underlying study aimed to establish an in vitro pre-screening platform to test treatment options’ effectivity and dosage, e.g., for new substances, autologous modified immune cells, or combined therapies in HCC. We initially employed 15 surgical resection specimens from patients with different HCC entities for isolation and preservation. The isolated liver cancer cells from four HCC-diagnosed patients were used for orthotopic transplantation into the healthy liver of immunodeficient mice, allowing them to grow for six months before human liver cancer cells were isolated and cultured. As a result, we generated and characterized four new primary-like liver cancer cell lines. Compared to immortalized HCC cell lines, freshly generated liver cancer cells displayed individual morphologies and heterogeneous protein-level characteristics. We assessed their ability to proliferate, migrate, form spheroids, and react to common medications compared to immortalized HCC cell lines. All four liver cancer cell lines exhibit strong migration and colony-forming characteristics in vitro, comparable to extensively investigated immortalized HCC cell lines. Moreover, the four etiological different liver cancer cell lines displayed differences in the response to 5-FU, Sorafenib, Axitinib, and interferon-alpha treatment, ranking from non-responders to responders depending on the applicated medication. In sum, we generated individual patient-derived liver cancer cell lines suitable for predictive in vitro drug screenings and for xenograft transplantations to realize the in vivo investigation of drug candidates. We overcame the low cultivation success rate of liver cancer cells derived from patients and analyzed their potential to serve a pre-clinical model.

## 1. Introduction

Hepatocellular carcinoma (HCC) accounts for over 90% of primary HCCs and is the fourth leading cause of cancer death worldwide [1]. Chronic liver disease, including cirrhosis, is the most relevant risk factor for HCC, mostly due to viral hepatitis, while excessive alcohol intake and obesity are the leading risk factors worldwide [2]. Virus-induced HCCs arise from chronic liver diseases caused by hepatitis B virus (HBV) or hepatitis C virus (HCV) infection, and a high incidence has historically been observed in Asia and Africa due to the high prevalence of these viruses in the local population [3,4]. Moreover, increasing numbers of HCC patients have been observed in most industrialized countries, which is related to the high incidence of obesity and underlying metabolic diseases known as non-alcoholic fatty liver diseases (NAFLDs) [5] and its more progressive form of non-alcoholic steatohepatitis (NASH), which is accompanied by hepatic inflammation and appears to be the leading cause of liver transplantation [6]. It has been shown that the incidence of developing an HCC is generally higher in males than in females due to their high testosterone levels [7]. HCC is a highly heterogeneous disease in terms of morphology, cellular behaviour, responses to treatment, and clinical outcome [8].

To elucidate the cellular and molecular liver-cancer-cell-related responding and non-responding cell agents during drug screening, it becomes increasingly important to reflect the heterogeneity of clinical HCC in the investigated cell lines [9]. As shown for other tumor entities (e.g., colon, breast, CNS, and prostate), the drug screening success rate was significantly higher when using patient-derived models [10]. These results lead to the retirement of old and extensively used cell-line-based screening panels, like NC-60 [10].

In HCC patients, individualized T-cell therapies, in combination with checkpoint inhibitors, may serve as a future therapeutic agent. Here, ex vivo models can enable a similar response to therapeutic agents as seen in patients with grown tumors. These ex vivo models are more suitable for reflection of the clinical samples than the most widely used immortalized cell lines for several reasons: (i) genetic aberrations of immortalized cell lines limit their usefulness and impact the final overview [11]; (ii) patients with identical genetic mutations tend to react differently to the same treatment options [12]; and (iii) accessing only a limited number of liver cancer cell lines makes it challenging to encompass the extensive heterogeneity of genetic and epigenetic variants found in millions of patients. Hence, there is a growing interest and a clinical need to establish primary liver cancer cell lines to reflect the natural microenvironment and to preserve characteristic crosstalk between cancer cells [13]. Procedures such as core biopsies, fine-needle aspirates, surgical resections, or autopsy specimens can yield patient-derived tissue samples for generating primary cell lines [11,14]. The difficulty in establishing primary liver cell lines lies in cultivating these cells for a long-term period since primary cells undergoing apoptosis or necrosis or primary cultures are contaminated by overgrowing fibroblasts [15].

Here, we employed a xenograft mouse model to overcome this limitation and give a better understanding of the effectiveness of drug candidates. Taking animal welfare, costs of mouse experiments, and dose efficiency evaluation into account, in vitro pre-screenings are still the gold standard. Our goal was to generate relevant liver cancer cell lines that can be used for both in vitro and in vivo drug testing. Therefore, we employed an adapted multi-fractional protocol combined with an orthotopic transplantation method for HCC cells derived from patients, aiming to cultivate those primary HCC cell lines [16] and to analyze their ability to be suitable for pre-clinical investigations.

This article will highlight the isolation, transplantation, and preservation of patient-derived individual cells by focusing on their cellular characteristics, surveillance, and usage in treatment application, and xenograft transplantation compared to extensively investigated established hepatocellular cell lines.

## 2. Methods

### 2.1. Isolation of Liver Cancer Cells from Patient- and Mouse-Derived Material

The foundation of this study is based on the isolation and culturing of human liver cancer cells from patients and mice. Liver tissue samples of various sizes and etiologies were available from 15 patients undergoing surgery at the Center of Surgical Medicine and the Clinic and Polyclinic for General, Visceral, and Thoracic Surgery at the University Medical Center Hamburg-Eppendorf (ethical study approval number PV3578; amendment for this study was approved 18 June 2020; recruitment started from 1 March 2021 and is still ongoing). The liver tissue was received in Belzer UW Cold storage solution (Bridge to Life, Northbrook, IL, USA) and was immediately stored on ice until further processing. Fresh liver and tumor material were prepared on ice with sterile scalpel blades (C. Bruno Bayha GmbH, Tutlingen, Germany) and forceps sterilely under a laminar flow hood. Adipose tissue, blood vessels, and necrotic areas were trimmed away. In preparation for enzymatic digestion, the tissue was transferred to a 6-well plate and minced into 1–2 mm^3^ pieces. The tissue pieces were then resuspended in DMEM + 100 IU/mL Penicillin und 100 µg/mL Streptomycin (1% P/S; Gibco, Paisley, GB and Grand Island, NY, USA) and immediately transferred to a MACS C-tube containing human tumor dissociation enzymes according to the manufacturer’s instructions (Tumor Dissociation Kit human). Tumor dissociation was performed using the gentleMACS^TM^ Octo Dissociator with Heaters, an appropriate liver-dissociation program, and constant rotating at 37 °C for 1 h. The cell suspension was then applied to a MACS SmartStrainer (70 µm). Kit, C-Tube, strainer, devices, and programs were purchased from Miltenyi Biotec (Bergisch Gladbach, Germany). Any remaining tissue residues on the strainer surface were carefully rubbed through the membrane using the rough side of a 5 mL syringe punch (BD, Heidelberg, Germany) and washed with 5 mL DMEM + 1% P/S. For cultivation, the SmartStrainer is placed up-side-down on a 6-well plate (Sarstedt AG & Co. KG, Nümbrecht, Germany) and remaining cells were rinsed off with HCC culture medium (Advanced DMEM/F-12 + 2 mM GlutaMAX™ + 1% P/S (Gibco^®^, Paisley, GB and Grand Island, NY, USA) + 1 µg/mL EGF (Miltenyi Biotec, Bergisch Gladbach, Germany) + 20% HyClone™FetalClone™II (Cytiva, Marlborough, MA, USA) and collected for cell culture using a T25 cell culture flask (Sarstedt AG & Co. KG, Nümbrecht, Germany). Additionally, Zellshield (Minerva Biolabs GmbH, Berlin, Germany) was added in a ratio of 1:100. The previously filtered cell suspension was centrifuged at 50× *g* at 4 °C for 10 min. The 50× *g* supernatant was centrifuged at 300× *g* at 4 °C for 10 min; the 300× *g* supernatant derived from human tissues was harvested and used for CD45-positive cell isolation (TILs, tumor-infiltrating leukocytes). A murine cell suspension containing transplanted human tumor cells was centrifuged at 700× *g* for 10 min (4 °C); the 700× *g* supernatant was discarded. Red blood cell lysis of 50× *g*, 300× *g*, and 700× *g* cell pellets was performed by means of 10 mL ACK-buffer (solved in Aqua dist.; 1.5 M, Ammonium chloride (NH_4_Cl), 100 mM Potassium bicarbonate (KHCO_3_), and 10 mM Titriplex III (EDTA-Na^2^)) at 37 °C for 5 min. Lysis was stopped by diluting the cell suspension with an equal amount of DMEM + 1% P/S. The Neubauer counting chamber and Trypan blue staining (1:5) determined the cell amount of each solution. Cells of each fraction (50× *g*, 300× *g*, and 700× *g*) were cultured in the HCC culture medium as previously described. Orthotropic transplantation into mice was performed by using 1 × 10^6^ cells per mouse derived from human or murine cell isolation. FACS characterization of HCC cells derived from human and murine cell isolation acquired 6 × 10^5^ cells of the 300× *g* fraction for staining, as described in Section 2.4. For long-term storage in liquid nitrogen, at least 1 × 10^6^ cells/mL of each fraction were prepared using a freezing medium (70% Belzer UW Cold storage solution, 20% FCS, and 10% DMSO (Sigma, St. Louis, MO, USA)) and Corning™CoolCell™ freezing container (Thermo Fisher Scientific, Waltham, MA, USA) for controlled freezing.

### 2.2. PBMC Isolation of Patient-Derived EDTA Blood

Peripheral blood mononuclear cells (PBMCs) were isolated from fresh patient-derived EDTA blood. The blood volume was determined and equally diluted for gradient centrifugation with cold RPMI (Gibco, Paisley, GB and Grand Island, NY, USA) without supplements. Diluted blood was then layered carefully on top of 15 mL Ficoll-Paque^®^ PREMIUM (Cytiva, Marlborough, MA, USA) and centrifuged without a brake at room temperature for 18 min. After centrifugation, the upper layer (plasma) was collected and stored at −80 °C. The PBMC layer underneath was carefully removed from the Ficoll layer by using a 1 mL pipette. PBMCs were washed with RPMI two times, counted, and archived in 1 mL Kryo safe medium I (c.c.pro, Oberdorla, Germany) per tube.

### 2.3. Protein Analysis by Immunofluorescence and ELISA

Human serum albumin (HSA) levels in human serum were measured using the human Albumin ELISA quantitation kit (Bethyl Laboratories, Biomol GmbH, Hamburg, Germany) according to the manufacturer’s instructions. Alpha Feto protein (AFP) levels were detected using the human alpha Fetoprotein Elisa Kit (R&D Systems, Minneapolis, MN, USA) according to the manufacturer’s instructions.

### 2.4. Flow Cytometry Characterization of Isolated HCC Cells

Isolated human HCC cells derived from fresh human liver cancer tissue, as well as from murine livers after injection of primary tumor cells, were characterized using the following monoclonal antibodies (also see Supplementary Figure S1 for the strategy): anti-CD13, anti-CD133-1, anti-CD24, anti-epithelial cell adhesion molecule (EpCAM), anti-human leukocyte antigen G (HLA-G), anti-CD90, anti-CD73, anti-CD105, anti-CD44, and anti-CD45, conjugated with phycoerythrin (PE), PE-Vio 615, PE-Vio 770, APC, and APC-Vio 770. Cells were stained in 1× DPBS containing 0.5% bovine serum albumin fraction (BSA, pH 7, GE Healthcare, Pasching, Germany) and 0.05% sodium azide NaN_3_ (Sigma, Steinheim, Germany). The staining was divided into five antibody panels to avoid overlapping emission spectra. Each panel requires 1 × 10^4^ cells. Positive cells were distinguished from negative cells using unstained samples. Live/dead staining was performed in advance in 1× DPBS (Gibco, Paisley, UK) using Viability Dye 405/520 Fixable Dye according to the manufacturer’s specifications. FACS measurement was carried out on the MACSQuant^®^ Analyzer 10 Flow Cytometer. All antibodies, Viability Dye, and Flow cytometer were received from Miltenyi Biotech (Bergisch Gladbach, Germany).

### 2.5. Generation, Breeding, and Housing of Immunodeficient Mice

In this study, we employed parental female and male mice with transgenic NOD.Cg.*Prkdcscid Il2rgtm1Wjl/SzJ* background (Strain #:005557) from Jax Laboratory. Parental females and males were used to develop a breeding colony within the LIV animal facility in Hamburg. Mice individuals were employed for the study at a starting age of 12 weeks. After xenotransplantation, mice were visited daily. Weight was measured three times a week. The individual mice were marked and identifiable using ear punches or tail marks. Mice were housed in individually ventilated cages (IVCs) to exclude infection or transmission of infectious diseases. IVCs were changed as described in the standard operation protocol of animal husbandry.

### 2.6. Surgery, Recovery Phase, and Observation of Mice

To achieve this study’s goal, we employed three animals for the initial transplantation of fresh or frozen human tumor cells for each tumor or cell line. The mice were anesthetized with isoflurane in all subsequent preparatory and surgical procedures. In the first step, the animal was injected subcutaneously with the painkiller metamizole (200 mg/kg). In the next step, a hair trimmer removed the abdominal hair from the animals. The shaved abdomen was then cleaned and disinfected with Betadine. This first cleaning took place away from the operating table. After the onset of action of the painkiller metamizole (20–30 min), the animal ws now fixed on the operating table, and the abdomen was cleaned and disinfected two more times with Betadine. The operating table was tempered at 37 °C to prevent the animals from cooling. A laparotomy was performed along the midline over 1–2 cm. After imaging the left lateral lobe of the liver, intrahepatic injection of the tumor cells followed. The cell solution (0.5–1 × 10^6^ HCC tumor cells dissolved in sterile Matrigel) was applied in a standardized volume of 20 µL utilizing a very thin injection cannula (30-gauge, 0.3 mm). Possible bleeding was quenched by absorbable hemostatic. After closure of the muscles using a non-resorbable filament and skin using two clips, the mouse was separated from the isoflurane anesthesia and was transferred to a conventional cage. Postoperatively, mice received carprofen (5 mg/kg subcutaneously) every 24 h for 72 h. The painkiller was stopped after 72 h. Subsequently, the mice were examined 1–3 times daily to detect possible pathological changes. Pathological criteria included anemia (inspection of tail color), signs of local infection, splenomegaly (palpation), and weight loss (weighing two times a week), as well as apathy and motor deficits (for example, sluggishness or pulling a limb).

### 2.7. HCC Cell Culture

Immortalized HUH-7, Hep3B, and HepG2H1.3 cells were maintained as described previously [17,18]. Isolated primary and mouse-derived human cancer cells were passaged in Advanced DMEM/F-12 with non-essential amino acids and 110 mg/L sodium pyruvate, supplemented with 1% penicillin-streptomycin (P/S), 2% glutamine, 20% FetalCloneTM II Serum (all received from Thermo Fisher Scientific, Waltham, MA, USA) and 10 ng/mL human EGF (Miltenyi Biotech, Bergisch Gladbach, Germany) after forming a monolayer of 70–80% density. Immortalized cell lines were cultured in DMEM containing L-glutamine and glucose, supplemented with 1% P/S, 10% Gibco fetal bovine serum (FBS; all from Thermo Fisher Scientific, Waltham, MA, USA). Cell lines were detached in Detachin™ Cell Detachment Solution (AMSBIO, Abingdon, UK) and cultured in a humidified atmosphere at 37 °C and 5% CO_2_.

### 2.8. Stable Integration of LeGO-iG2-Puro+-Luc2 Construct by Lentiviral Transduction

To use bioluminescence imaging, luciferase (Luc, *Photinus pyralis*) expressing tumor cells were generated. For this purpose, lentiviral transduction was performed. The vector LeGO-iG2-Puro+-Luc2 (3rd-generation HIV1-derived self-inactivating vector) [19,20] was used, expressing luciferase under control of the ubiquitous SFFV-promoter linked by an internal ribosome entry signal (IRES) to a fusion protein consisting of eGFP, a 2A peptide, and puromycin N-acetyl-transferase, leading to resistance to puromycin. Primary HCC cell lines and immortalized HCC cells were stably transduced with this vector. For lentiviral transduction, 3 × 10^5^ cells per well of indicated tumor cell lines were plated in 6-well plates. After 24 h, viral particles were added to gain a multiplicity of infection (MOI) of 10 and 100. The transduced tumor cells were selected by adding 0.5 µg/mL puromycin to the appropriate regular culture medium. Transduction efficiency was verified via eGFP fluorescence signal using flow cytometry.

### 2.9. In Vivo Imaging of Mice by Determining Luminescence Signal

Monitoring tumor growth started one week after transplantation and was assessed using bioluminescence after intraperitoneal injection of D-luciferin (30 mg/mL). After induction of general anesthesia, D-luciferin was injected accordingly to the weight of the mouse (150 mg/kg), and mice were imaged after 9 min with an IVIS-200 imaging system (PerkinElmer, Waltham, MA, USA). Post-processing and quantification of the emitted photons was performed using the Living Image software 4.7.4 (PerkinElmer, Waltham, MA, USA). For each mouse, a quadratic region of interest (ROI) encompassing the primary tumor was selected, and the luminescence in each ROI was acquired in total flux (p/s).

### 2.10. Protein Analysis by Western Blot

For protein extraction, cell pellets were incubated in RIPA buffer (solved in Aqua dest; 65 mM Trism base, 1% Nonidet P40, 154 mM NaCl, 0.1% Sodium dodecyl sulfate SDS, 1 mM EDTA) and 1× Protein inhibitor cocktail (PIC, Merck KGaA, Darmstadt, Germany) on ice, for 30 min. Samples were then centrifuged at 14,000 RPM, 4 °C, for 30 min. The BCA Protein Assay Kit (Thermo Scientific, Rockford, IL, USA) was used to determine protein concentrations in the supernatants. Proteins were diluted to 30 ng/mL with NuPAGE LDS sample buffer 4× (Thermo Fisher Scientific, Waltham, MA, USA) and RIPA + 1 × PIC. Denaturation took place at 75 °C for 10 min. Protein separation by SDS-PAGE using NuPAGE 4–12% Bis-Tris gels and XCell SureLock Mini Cell, as well as the transfer to PVDF membranes through the iBlot™ 2 Dry Blotting System, was carried out according to the manufacturer’s instructions (Thermo Fischer Scientific, Newport, UK). Separation required 130 V for 1 h and 50 min. PVDF membranes were probed with specific antibodies and detected by enhanced chemiluminescence (SuperSignal™ West Pico PLUS, Thermo Scientific, Waltham, MA, USA). The specific antibodies used were TP53 antibody (FL-393) HRP (sc-6243; Santa Cruz, Dallas, TX, USA) and GAPDH loading control monoclonal antibody (GA1R) HRP (#MA5-15738-HRP; Thermo Fischer Scientific, Newport, UK).

### 2.11. Protein Analysis by Immunofluorescence

For ex vivo immune histological identification of specific marker CD44 and CK19, tissue slides derived from patients were treated with 4% PFA for up to 24 h at 4 °C and embedded in paraffin. For staining, slides were placed onto positive-charged microscopic slides (Agilent, Waldbronn, Germany) and incubated at 60 °C for 30 min. Immunohistological staining was performed on an automated Bond III immunostaining system (Leica Biosystems, Wetzlar, Germany) using anti-CD44 and anti-CK19 antibodies (see Supplementary Table S1). The slides were rinsed with an SDS-based buffer (Leica Biosystems) between each incubation step. Antigen-dependent epitope retrieval was conducted at 100 °C using basic EDTA-based buffer (Leica Biosystems) for 10 min. After blocking endogenous peroxidases by applying 3% hydrogen peroxide solution, primary antibodies were incubated at room temperature for 13 min. Detection was performed using an HRP-conjugated polymer/DAB-based detection kit (Leica Biosystems) according to the manufacturer’s recommendations. Nuclei were counterstained with hematoxylin. An appropriate positive control was carried along each run. For ex vivo fluorescence immunological (IF) staining of cryopreserved mouse-derived liver tissue, slides were fixated using 4% PFA for 10 min. PFA-fixed cryosections were stained with monoclonal anti-CK19, monoclonal anti-Ki67, polyclonal anti-CD44, and monoclonal anti-HLA-ABC (Supplementary Table S1). For in vitro histological characterization, 10,000 cells were seeded into 96-well plates and were allowed to reach 80% confluence, when they were washed with 1× PBS and fixe with 4% PFA for 10 min. After fixation, mouse-derived tissue slides and generated LC cells were used for immunofluorescence staining against indicated proteins using primary antibodies, as in Supplementary Table S1. Specific signals were visualized with Alexa 488 or 555 labeled secondary antibodies (Invitrogen, Darmstadt, Germany). Nuclear staining was achieved by Hoechst 33258 (Invitrogen, Eugene, OR, USA). Stained cells were analyzed by fluorescence microscopy (BZ-9000 and BX-780, Keyence, Osaka, Japan). Captures of tissue slides were generated manually with consistent exposure times for equal material and antibodies. For LC cells, captures were generated automatically with same magnifications and exposure time for same antibodies.

### 2.12. Doubling Time Assay

For cell doubling, xCELLigence RTCA SP device (ACEA Biosciences) was measured by seeding 10,000 cells into a E-Plate 16 (16-well plate) and allow them to grow for 72 h in HCC culture medium (pHCC cell lines) or in DEMEM (Huh-7, HepG2H1.3, Hep3B). Doubling time was calculated from 5 technical replicates and by the usage of the Xcelligence RTCA Software v2.0 for real-time data analysis [21].

### 2.13. Colony-Formation Assay in Matrigel

Cell proliferation was evaluated by average size and number of 3D tumor colonies under anchorage-independent growth conditions in 5 mg/mL Matrigel (MG; Corning Incorporated, NY, USA). Therefore, as explained above, a 96-well plate (Sarstedt AG & Co. KG, Nümbrecht, Germany) was coated with MG diluted in the appropriated culture medium. After solidifying at 37 °C, the upper layer of an MG cell suspension (38 cells/well, in a final volume of 53 µL cell MG suspension each well) was plated as described above. The plate was then incubated for 45 min at 37 °C and finally augmented with the adequate culture medium. The colony formation took 11 days of incubation at 37 °C and 5% CO_2_. A change in the medium was performed after 7 days. For the evaluation of colony number and surface area, images were captured using Zeiss Axiovert 35 and Zeiss Axiocam ERc 5s (Carl Zeiss, Jena, Germany) of colony number and surface area. ZEISS ZEN lite 3.5 (blue version) software (Carl Zeiss Microscopy, Oberkochen, Germany) was used to photograph the colonies at 10× magnification. The colony area was determined in µm^2^ by ImageJ 1.53 (NIH, NY, USA).

### 2.14. Colony Formation on PolyHema

To investigate the colony-forming properties of indicated cells lines, a round-bottomed 96-well plate was coated with 12 mg/mL PolyHema (Poly (2-hydroxyethyl methacrylate; Sigma, St. Louis, MO, USA) dissolved in 95% EtOH (Chemsolute, Renningen, Germany). Then, 1 × 10^5^ cells per well were seeded in the appropriate culture medium, see Section 2.1 and centrifuged at 100× *g* for 5 min. Cells were incubated in a humidified atmosphere at 37 °C and 5% CO_2_ for 10 days. Colony morphology was recorded by taking images, as described in Section 2.13. Medium change was performed as required.

### 2.15. Migration Assay

First, 3.5 × 10^5^ cells of indicated cell lines and primary-like cell lines were seeded into 12-well plates (Thermo Fisher Scientific, Waltham, MA, USA) and allowed to grow until confluency. Cells at confluence were scraped off, creating a thin cell-free area using a sterile 200 µL pipet tip. After scratching, the medium was renewed, excluding detached cells from the culture. To analyze the migration of the cells, a reference image centered on the scratch was taken from each well as described in Section 2.13. The images were acquired using a 10x magnification every 24 h for 4 days, including day 0 (the day of the scratch) or until gap closure, repectively. Between observations, the culture plates were incubated in a humidified atmosphere at 37 °C and 5% CO_2_ in an incubator. Using the image-processing software ImageJ 1.53 (NIH, New York, USA), the gap surface of 3 to 4 wells was determined in µm^2^ and finally specified in relative migration in %.

### 2.16. Treatment Experiments

For toxicity experiments, 10,000 cells were seeded per well of a 96-well E-Plate; after 24 h, cells were treated for 72 h with chemotherapeutic 5-FU or kinase inhibitors Sorafenib and Axitinib using indicated concentrations ranking from 30 µM to 0.014 µM. For BrU-based proliferation assays to access the effects of the treatment on newly synthesized DNA, 100,000 cells were seeded into 24-well plates and were treated with effective or highest concentrations of 5-FU (0.1–0.3 µM), Sorafininb (10–30 µM), or Axitininb (10–30 µM) after 24 h, for 72 h. For protein and gene expression analysis, 150,000 cells were seeded into 24-well plates and were incubated with interferon-alpha (100 IU/mL) after 24 h for 18 h.

### 2.17. Analysis of Toxicity Level after Treatment

Cell viability was measured after 72 h of incubation with 5-FU, Sorafininb, or Axitinib using (3-4, 5-Dimethylthiazol-2-yl)-2, 5-diphenyltetrazolium bromide (MTT; Invitrogen) according to the manufacturer’s instructions.

### 2.18. Analysis of Proliferation Level after Treatment

Newly synthesized DNA was detected using the Click-iT EDU Imaging Kit Alexa Fluor 555 (Invitrogen) according to the manufacturer’s instructions. Previously, 100,000 cells were seeded per well of a 24-well plate. After 24 h, the cells were treated with indicated substances at indicated concentrations for 72 h. Twelve analysis fields were captured and counted out of two wells using the same conditions. Representative output data is shown in Supplementary Figure S2A–C.

### 2.19. Analysis of Surface Marker Alternation after Treatment

Treated cells were characterized using the following monoclonal antibodies: anti-HLA-ABC (Invitrogen, Waltham, MA, USA), anti-PD-L1 (Becton Dickinson, Franklin Lakes, NJ, USA), and anti-HLA-DR (Becton Dickinson, Franklin Lakes, NJ, USA), conjugated with PE, Brilliant Violet 421. The staining was divided into two panels to avoid overlapping of emission spectra. Positive cells were distinguished from negative cells using unstained samples. FACS measurement was performed on the BD FACSCelesta Cell Analyzer.

### 2.20. Isolation of Oligonucleotides

RNA was extracted from human liver specimens using the RNeasy Mini RNA purification kit (Qiagen GmbH, Hilden, Germany). RNA extracted from human-derived cells and generated HCC cell lines using the RNeasy RNA Micro purification kit (Qiagen GmbH, Hilden, Germany).

### 2.21. Measurement of Gene Expression Level

For measurement of gene-expression, 2-step PCR was performed. Therefore, cDNA synthesis was conducted by using MMLV Reverse Transcriptase 1st-Strand cDNA Synthesis Kit (Lucigen, Middleton, WI, USA) to synthesize RNA complementary DNA, according to the manufacturer’s instructions. Human-specific primers from the TaqMan Gene Expression Assay System were used to determine gene expression levels (Life Technologies, Carlsbad, CA, USA). Samples were analyzed with the Quant Studio 7 Real-Time PCR System (Life Technologies, Carlsbad, CA, USA). The mean of the human housekeeping genes GAPDH and ribosomal protein L30 (RPL30) was used to normalize human gene expression levels. Specific probes used for gene expression analysis are listed in Supplementary Table S2.

### 2.22. Statistics

For graph design and statistical analysis, the analysis software GraphPad Prism Version 9 (GraphPad Software, Inc., La Jolla, CA, USA) was used. Doubling time comparison shown in Figure 2C were performed using one-way ANOVA analysis followed by multiple comparison test against immortalized cell lines or untreated control. TP53 expression comparison (Figure 3D) was analyzed using a two-tailed unpaired *t*-test and 3 technical replicates. Statistical comparison of more than two groups of biological triplicates was calculated using one-way ANOVA and post hoc Tukey’s multiple comparison test and plotted in Figure 5A–C,F. Treatment comparison shown in Figure 6D–F were performed using one-way ANOVA analysis followed by multiple comparison test against immortalized cell lines or untreated control. Plotted bar charts represent mean ± SD of technical multiple replicates. Treatment comparison for INF alpha, plotted in Figure 6H–J, was analyzed using a two-tailed unpaired *t*-test and 3 technical replicates. Statistical outputs are indicated in the figure legends; *p*-values were plotted in the graph as follows: * *p* < 0.05; ** *p* ≤ 0.01 and *** *p* ≤ 0.001, **** *p* ≤ 0.0001.

## 3. Results

### 3.1. Complete Preservation of Patient-Derived Cells from Resections and Blood by Applying a Multi-Fractional Dissociation Protocol

**Figure 1:**
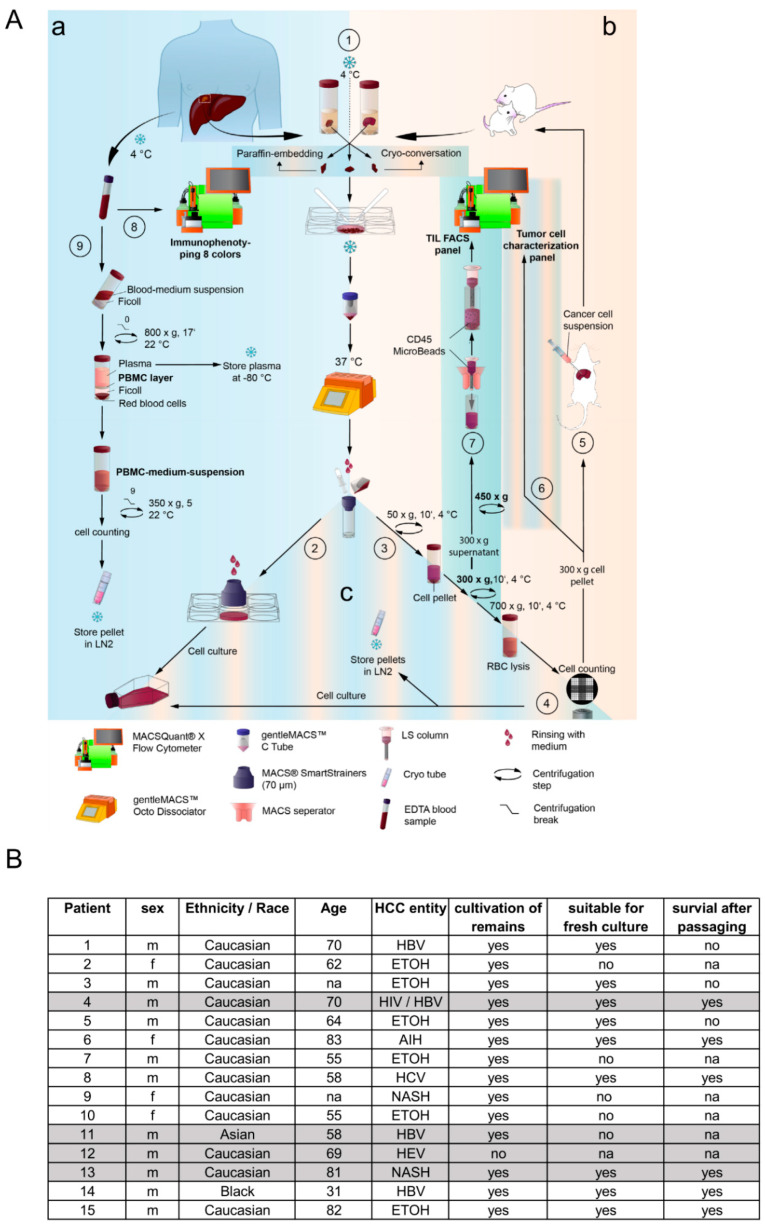
(**A**) Workflow of human primary-like HCC cell isolation from human (**a**) and murine liver tissue (**b**) and patient characteristics (**B**). (1) Sections of tumor material were used for cryo-conversation, paraffin-embedding, and cancer cell isolation. Tumor cell dissociation of patient-derived liver cancer tissue or murine orthotropic primary-like HCC cell-transplanted liver tissue (step 5) by mincing and transferring the tissue into a gentleMACS™ C Tube, prepared with an enzyme mix and using a GentleMACS™ Octo Dissociator with heaters and continuously rotation, followed by filtering the cell solution through a 70 μm MACS SmartStrainer. (2) Cultivation of cells retained on the strainer. (3) Serial centrifugation of the throughput (step 1) referring to human primary-like HCC samples (50× *g*, 300× *g*) and mice-derived material (50× *g*, 300× *g*, 700× *g*), followed by an RBC (red blood cell) lysis of cell pellets. (4) RBC-lysed suspensions were counted for storage and culturing. (5) Orthotropic transplantation of tumor cells into mice was accomplished by an intrahepatic injection of cells derived from the 300× *g* cell suspension. (6) Tumor cell characterization by flow cytometric measurement. (7) TIL isolation and characterization from human primary-like HCC samples by means of magnetic CD45-MicroBead labeling and MACS LS column, placed on a magnetic MACS separator followed by flow cytometric measurements for immune-system-specific surface markers. (8) Determination of immune cell populations in patient-derived EDTA blood by flow cytometry, using an 8-color Immunophenotyping kit. (9) PBMC isolation by means of density gradient centrifugation. Briefly, diluted EDTA blood was layered carefully on top of FICOLL^®^ and centrifuged at 800× *g* without a break, using low acceleration. Plasma was collected and stored at −80 °C. PBMCs were counted and stored in LN2. CSS (cold storage solution); HCC; RBC (red blood cell); × *g* (gravity, centrifugation force); TIL (tumor-infiltrating lymphocytes); EDTA (ethylene diamine tetraacetic acid); PBMC (peripheral blood mononuclear cell); LN2 (liquid nitrogene). (**B**) Assignment of patient data, including sex, race, age, final diagnosis, and the initial cultivation characteristics. The patient marked in grey have been chosen for orthotopic transplantation and were described in the underlying study.

This study establishes a robust protocol to completely isolate and preserve primary HCC tissue and associated immune cells from small, distinct resections and blood extracted from patients suffering from HCC. As visualized in Figure 1A (part a), the preservation starts directly after surgery by applying organ transport solution to the sample to inhibit apoptosis induction and degradation of the cells. Patient material was then partially cryo-conserved and paraffin-embedded, as shown in Figure 1A, to allow gene expression analysis, as displayed in Supplementary Figure S3A. This material can be used for further immunological analyses. Generally, the rest of the sample was stored in the organ transplant solution for 30–60 min before applying the isolation procedure (steps 1–4, 7–9). Figure 1A shows an adaptation of the human tissue dissociation protocol from Miltenyi by using the gentleMACS and the recommended enzymes, as described previously [16]. In addition to enzymatic dissociation, mechanical separation is vital in producing satisfactory results. Since the processed HCC resections generally appeared soft, we adapted the standard Miteny GentleMACS Protocol to these conditions. Therefore, we reduced the rotation step’s speed and frequency and elongated the enzymatic incubation step. While enzymatic and mechanical dissociation was performed, blood samples were used to isolate corresponding peripheral blood mononuclear cells (PBMCs) (Figure 1A, part a, steps 8–9). At the same time, we performed immunophenotyping with whole blood samples (results are not shown). PBMCs were cryopreserved (part c). As shown in Figure 1A, strainers were used to accomplish the mechanic separation step (2), and magnetic beads were used to isolate tumor-infiltrating lymphocytes (TILs) (part b, step 7). The heterogeneous dissociated liver tissue sample was further processed by fractional centrifugation to allow the separation of hepatocytes from cancer cells and non-parenchymal cells by size (Figure 1A, part c, step 3). After the final erythrocyte lysis, single fractions were cryopreserved and used for surface marker identification.

We collected undissolved tissue remains for 93% of all processed samples, as mentioned in Figure 1A part c, step 2, and tested cultivation under primary culture conditions. Under these conditions, 64% of the processed samples exerted an adhesive nature and formed 2D clusters, whereby two-thirds of these cells were also suitable for the detaching procedure. Since no feeder cells, proliferation-inducing factors, or immortalization was used in this study to support the proliferation of the obtained primary cultures, they appeared unsuitable for long-term culturing. We exemplarily employed 4 out of the listed 15 HCCs (Figure 1B) for orthotopic in vivo transplantation (marked in grey in Figure 1B) to analyze their ability to generate primarily derived cell lines. For transplantation into the immune-deficient mice, we employed cryopreserved material from patient 4 (HIV/HBV-induced HCC), patient 11 (HBV-induced HCC), patient 12 (HEV-induced HCC), and patient 13 (NASH-induced HCC). The four patients have been selected as their underlying diseases displaying a high clinical significance. As mentioned before the chronic HBV infection and the chronic coinfection HIV/HBV reflexes, one of the leading causes for HCC development. The rising incidence NASH in the Western world, as mentioned in extrapolations, was our reason to collect patient 13. We additionally employed patient 12, who developed an HCC with diagnosed chronic Hepatitis E virus infection. The HEV-related mortality in patients is racking from 0.5–4%, which displays a high variety and a low clinical relevance. In this regard, the influence of the chronic HEV infection in the progression of the HCC is not extensively studied and drug screenings mostly do not include this type of tumor [22]. Therefore, we aimed to close the gap and provide an HEV-derived HCC cell line.

The thawed batches of the patient’s material exert a median viability of 80% before transplantation. As a result, all four patient-derived cell fractions were suitable for repopulation of the mouse liver, since all four could be reisolated and cultured for the long term after 6 months of cultivation in the physiological environment.

### 3.2. The Repopulation of Patient-Derived Liver Cancer Cells in NSG Mouse Livers Alters the Presence and Distribution of Tumor Stem Cell Markers

**Figure 2:**
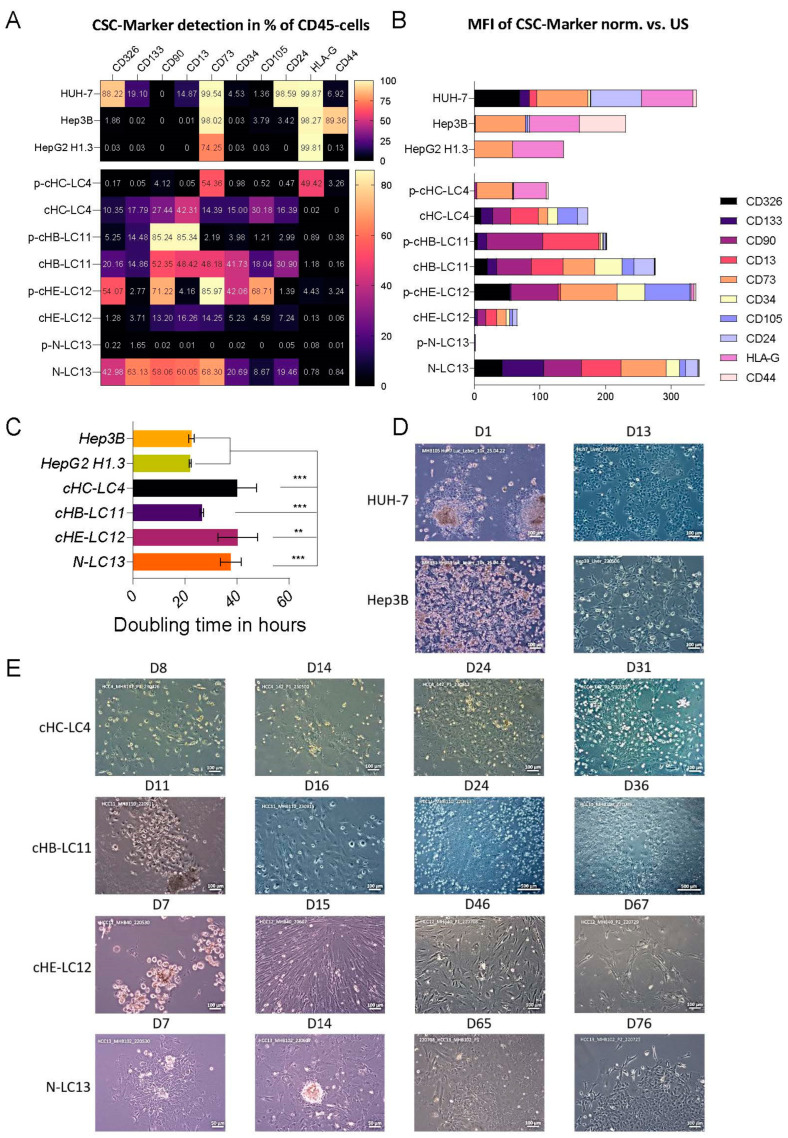
Cell type analysis and morphological documentation of isolated pHCC cells vs. immortalized cell lines. Regarding stem cell surface and tumor marker, different expression levels are shown in isolated pHCC cell lines and established cell lines. (**A**) Expression pattern of surface marker heat maps show expression summary of selected CSC surface marker (CD326, CD133, CD90, CD13, CD73, CD34, CD105, CD24, HLA-G, CD44) by flow cytometry of primary (p-N-LC13, p-cHB-LC11, p-cHIB-LC4, p-cHE-LC12), ex vivo isolated cells lines (N-LC13, cHB-LC11, cHIB-LC4, cHE-LC12), and established immortalized cell lines (HUH-7, Hep3B, HepG2H1.3). Heat maps show the percentage of living, CD45-negative fraction. (**B**) Determination of CSC-Marker MIF levels in stacked contingency bar charts, displaying frequencies for combinations between cell surface marker expression and indicated cell lines. Data are presented as the median of MFI. Each value represented a single measurement and was normalized against unstained controls for background elimination. (**C**) The doubling time assay was performed after pHCC cell lines were cultured under specified cell culture conditions, and division rates were compared to immortalized cell lines. (**D**,**E**) Show longitudinal photographs of isolated and cultured cells after orthotopic transplantation until cells reach monolayer structure. In (**D**), fresh isolated immortalized HUH-7 and Hep3B cell lines were observed for 13 days. In (**D**,**E**), fresh isolated pHCC cultures were documented in type-specific laps of time. Photographs were carried out in the same magnification from indicated cultures. Bar charts are displayed in hours and for n = 3 replicates per cell line. Since immortalized cell lines overcome the monolayer structure, the doubling time assay was performed by counting after 72 h and 144 h. Results were calculated as indicated in the method section. All statistical differences were characterized by plotting the *p*-value as follows: ** *p* ≤ 0.01 and *** *p* ≤ 0.001.

To support the proliferation competence of the four HCC cell isolates derived from patients 4, 11, 12, and 13, we mimicked their physiological environment by utilizing orthotopic transplantation into the large liver lobes of immune-deficient mice. HCC cells derived from all four patients (4, 11, 12, and 13), listed in Figure 1B (grey), were isolated again, but from the mouse liver six months after transplantation. Isolated fractions of the four human HCC-derived batches were compared to their mouse-derived isolated counterparts and to the immortalized cell lines Hep3B, HepG2H1.3, and HUH-7 by surface marker staining, to understand the tumor development processes, by assessing the occurrence of cancer stem cells (CSC) [23]. As shown in Figure 2A,B, primary HCC cells (p-cHB-LC11, p-cHIB-LC4, p-cHE-LC12, and p-N-LC13) exhibited a wider variety of CSC marker expression compared to immortalized HCC cell lines (Hep3B, HepG2H1.3, and HUH-7). As summarized in Figure 2B, CD73 expression was prominent on all analyzed cell lines, whereby immortalized cell lines HUH-7 and Hep3B displayed the highest levels of CD73 presentation, which aligns with the observed tumor progression in vivo (Supplementary Figure S4A,B). Here, the orthotopic transplantation of luciferase-producing HUH-7 and Hep3B cells into the liver of NSG mice resulted in rapid tumor formations leading to macroscopically visible tumors within 3 to 5 weeks post-operation, as shown in Supplementary Figure S4A,B. In comparison, luciferase-producing HCC cell line cHB-LC11 displayed distinct tumor formation within 15 weeks post-transplantation. HUH-7 cells prepopulate the liver, spleen, and pancreas. During that time, Hep3B and cHB-LC11 attempted to grow exclusively in the transplanted mouse livers.

The surface marker analysis showed that the immortalized cell lines are highly expressed HLA-G. For HepG2H1.3 cells, we detected positive signal staining only for CD73 and HLA-G markers. HUH-7 cells exhibited more CSC-like characteristics by expressing CD24, CD13, CD44, CD34, and CD133. Nevertheless, the highest expression levels were detected for CD73 and HLA-G besides CD24, followed by CD326. Hep3B cells represented high CD73, HLA-G, and CD44 expression levels, and low CD105 and CD24 levels. However, these data presented distinct cell types regarding immortalized cell lines.

For mouse-derived human HCC cell lines, we detected a wider variety of marker expression, as shown in Figure 2A,B. Here, for the three primary-derived cell lines cHIB-LC4, cHB-LC11, and N-LC13, passaging elevated the occurrence and the presentation of these CSC markers. For cHE-LC12, the percentage and occurrence of CSCs were reduced compared to parental cells. The parental cHIB-LC4 cells displayed high CD73 and HLA-G expression levels, while cHIB-LC4 cells expressed no HLA-G and reduced CD73 on the cell surface. On the other hand, the expression of all other markers was elevated. The parental cHB-LC11 cells were highly positive for CD13 and CD90 but exhibited low CD326, CD73, CD34, CD105, and CD24, and intermediate rates for CD133 expression, respectively. After adaptation to the mouse liver environment, we measured a highly heterogeneous cell population, whereby CD90, CD13, CD73, and CD34 expression was most prominent. Here, a small portion of the cells expressed HLA-G on the surface. Initially, less presented CSC markers were elevated by up to 20-fold. The presentation of the CSC marker CD 133 was consistent between parental and passaged cells.

Interestingly, parental N-LC13 cells were almost negative for all CSC markers. Only a tiny portion of the parental cells were positive for CD133. After performing the orthotopic transplantation, almost all CSC markers were expressed on the surface of the cells, partially at very high percentages.

### 3.3. Orthotopic Transplantation of Four HCC-Derived Primary Cell Batches Preserves Their Integrity and Enables Long-Time In Vitro Cultivation

The mouse livers, including human HCC cells, were maintained as described previously, allowing them to colonize and proliferate under primary cell culture conditions. As shown in the representative captures from ex vivo cultures in Figure 2D,E, primary cells exhibited adaptability to the extracellular matrix within the first weeks of cultivation by showing adhesive and clustering quality and, therefore, the ability to establish cell line characteristics. Compared to each other and to immortalized Hep3B and HUH-7 cells, the obtained primary cell lines presented different tempos and patterns of adaption. Successful colonialization events depended on cell–cell interactions between major cancer-associated fibroblasts (CAFs) and minor primary-like HCC cells. First, the adhesion of CAF took place. After that timepoint, CAFs migrated and started to form loose connections with themselves. Once this event appeared, HCC cells were attracted to migrate into the loose-formed structures and begin to create dense clusters. After setting up this interaction, primary-derived cells started to proliferate. The initial formation took place within the first two weeks after cultivation. After another week of cultivation, primary cultures appeared in more prominent spots, forming a monolayer structure with close junctions. After these formations were reached, the detaching procedure was performed, and primary cell cultures underwent passaging.

In comparison, immortalized cell lines displayed a different pattern of colonialization and proliferation after the isolation procedure. These cell lines exerted a rapid adaption of the extracellular matrix and started expansion after adhesion. As shown in Figure 2C, doubling times of primary-derived cells varied among each other and to immortalized cell lines. N-LC13 (cell line of patient 13) doubled after 22.7 h, like HepG2H1.3 and Hep3B cells (25 h), whereas cBH-LC11 (cell line of patient 11) and cHE-LC12 (cell line of patient 12) doubled after 55–60 h and cHIB-LC4 (cell line of patient 4) after 90 h.

As shown in Figure 2D, Hep3B and HUH-7 cells formed overlapping layers within two weeks of cultivation, whereby the dense cell–cell interaction was not necessary. After reaching passages 5–7, primary-derived HCC cell lines were used for characterization.

In summary, we overcame the limitation of cultivation for the primary-derived liver cancer cells received from patients 4, 11, 12, and 13 (Figure 1B) by passaging the primary isolates via the liver of immune-deficient mice. Moreover, after re-isolation, we observed a recurring process in migration and formation for the four employed cell batches in vitro. After migration and formation were performed by the two major cell types (CAFs and hepatocellular carcinoma cells), all four primary cell cultures developed a 2D monolayer structure and could be used as primary-derived HCC (pHCC) cell lines (cHB-LC11, cHE-12, cHIB-LC4, and N-LC13).

### 3.4. Generated HCC Line Characterization Shows Human Hepatocyte- and Cholangiocyte-like Cells Accompanied by Small Portions of Liver-Associated Cells

**Figure 3:**
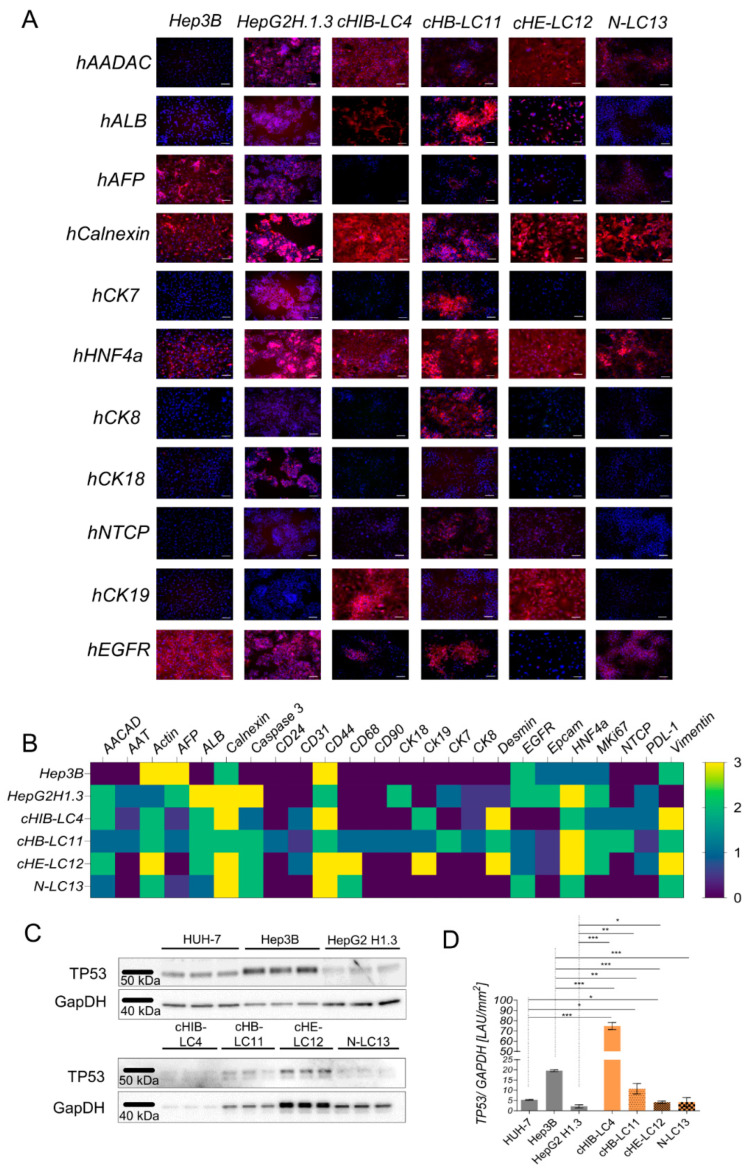
Protein-based characteristics of newly derived HCC cell lines compared to established cell lines. (**A**) shows immunofluorescent-based staining of indicated cell lines at single marker level for hepatocyte-specific proteins in red against the nuclei in blue. The scale represents 100 μm. (**B**) displays a quantitative analysis of the intensity and distribution of positively stained proteins in the indicated cell lines, ranking from 0 = negative to 3 = highly positive in almost all cells; 1 = positive in definite spots or plenty of single cells, and 2 = positive in whole areas. (**C**) p53 expression in ex vivo re-cultivated cells lines (N-LC13, cHB-LC11, cHIB-LC4, cHE-LC12) and established cell lines (HUH-7, Hep3B, HepG2H1.3) displayed in Western blot analysis. (**D**) Quantitative differences in p53 expression between indicated cell lines show statistically significant changes between established and isolated re-cultivated cell lines. All statistical differences were characterized by plotting the p-value as follows: * *p* < 0.05; ** *p* ≤ 0.01 and *** *p* ≤ 0.001.

We employed various human-specific markers for IF staining to characterize the four generated human liver cancer cell lines more in detail, as summarized in Supplementary Table S1. The aim was to differentiate the liver cancer cultures after passing them several times in vitro. As shown in Figure 3A, all four human liver cancer cell lines were positively stained for the hepatocyte-specific markers Calnexin and HNF4alpha, while other markers were differently expressed among each other. As shown in Figure 3B, the four generated cell lines were compared to immortalized cell lines Hep3B and HepG2H1.3.

As a result, we found the liver cancer cell lines cHB-LC11, cHE-LC12, and cHIB-LC4 to exert a hepatocyte-like phenotype, whereas the cancer cell line N-LC13 appeared to display a cholangiocyte-like phenotype.

In all four cell lines, we could not observe a positive expression of mouse-specific markers, as shown in Supplementary Figure S5A,B.

The clinical tumor marker alpha-feto-protein (AFP) was detectable at low protein levels in three out of four of the primary human liver cancer cell lines. For cHIB-LC4, no production of AFP was detectable on the protein level. This finding aligned with the patient’s serum protein levels on the day of resection, as shown in Supplementary Figure S5C. Here, AFP was highly expressed in patients 11, 12, and 13, but low in patient number 4. At the same time, we found the human serum albumin level (ALB) significantly reduced in all patients compared to the healthy control group, as shown in Supplementary Figure S5C. Nevertheless, the protein expression level of ALB in the four liver cancer cell lines displayed the same pattern as observed in the patients. These findings indicate that the generated human liver cancer cell lines exhibit protein expression similarities to the parental material.

**Figure 4:**
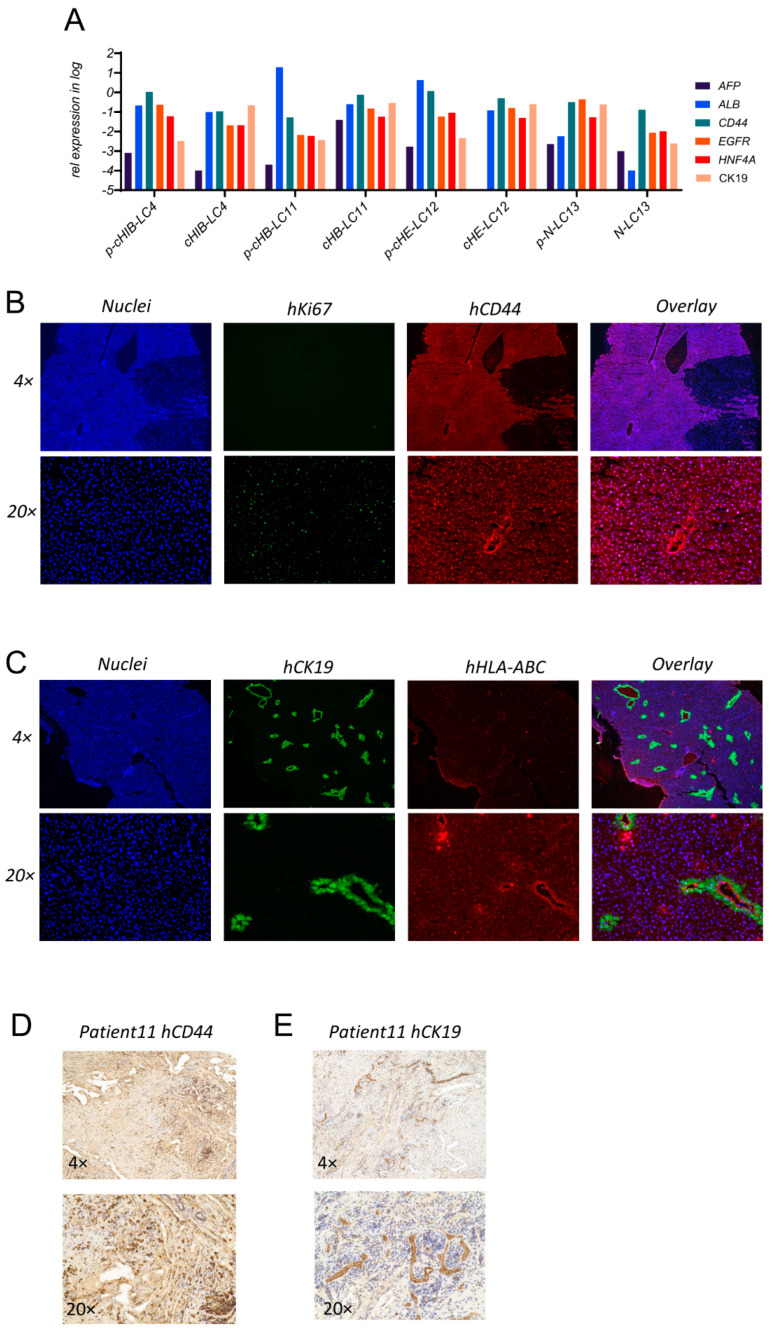
Cell-type-specific comparison of patient’s tumor tissue slides and isolated tumor cells with generated LC lines in vitro and in vivo. (**A**) Human gene expression levels of cell-type-specific genes (AFP, ALB, CD44, EGFR, HNF4A, CK19) are shown measured from patient-derived isolated cell fractions (p-HCCs) in comparison with orthotopically generated cell lines (LCs). Gene expression levels were normalized against continuously expressed housekeeper genes RLP30 and GapDH. (**B**,**C**) IF staining from representative tissue slides of patient-derived tumor sections is shown. In (**B**,**C**), fluorescence staining of mouse livers transplanted with cHB-LC11 cells is displayed in indicated magnifications, in single and merged detections of the particular fluorescence dye. In (**B**), human anti-CD44 is visualized in red in combination with the proliferation marker MKI67 in green. In (**C**), human anti-CK19 in green is displayed in combination with MHC-class I binding antibody anti-HLA-ABC in red. Nuclei were detected by Hoechst staining and visualized in blue. In (**D**), parental tumor tissue derived from patient 11 (HCC11) was stained for CD44 and shown in indicated magnifications. In (**E**), human anti-CK19 is shown in the same comparison pattern and magnifications.

In all four liver cancer cell lines, the tumor progression marker CD44 [24] was highly prominent, in contrast to the cytometer-based surface marker analysis, but in line with the gene expression analysis shown in Supplementary Figure S3 and in Figure 4A. CD44 was detectable by IF staining in the nucleoplasm and the cytoplasm in the 2D cell culture, and, moreover, in the IHC and IF staining of liver tissue slides from patients and mice, as shown in Figure 4B,D. The human-specific hepatocyte marker cytokeratin 18 (CK18) was expressed exclusively in HepG2H1.3 and cHB-LC11 cells. Cytokeratin 19 (CK19), which is associated with a poor prognosis and aggressive behaviour in human HCCs [25], was produced in cHB-LC11, cHE-LC12, and cHIB-LC4 cells but not in N-LC13 cells or immortalized HCC cells.

As shown in Figure 3B, primary-derived liver cancer cell lines comprise different cell types. Here, we analyzed CD31, PDL-1, CD24, CD90, CD68, and EpCam as cell type specific markers. Few CD31-positive sinusoid cells have been detected in all three virus-induced generated liver cancer cell lines. At the same time, no expression was detectable in the immortalized cell lines, and the NASH-derived generated cell line. The immune-suppressive protein PDL-1 was also exclusively detectable in virus-induced, and virus-producing analyzed cell lines. The occurrence of PDL-1 suggests its potential role in immune evasion and modulation. Only the HBV-related cHB-LC11 cell line exhibited the presentation of CSC marker CD90 and CD24 at the same time. This finding was in line with the acquired FACS analysis (Figure 2A), which revealed the highest expression of CD24 and CD90 in cHB-LC11 cells, except N-LC13 for CD90, which appeared to be equally expressed in the cytometry analysis but was not expressed to that extent in long-term culture. The macrophage marker CD68 was highly expressed on cHB-LC11, cHE-LC12, and N-LC13 cells, but appeared to not be expressed in the other cell lines in vitro. It is worth mentioning that the presentation of the mentioned marker was detectable in a few or even single cells in the obtained cell lines, with the acceptance of CD68 in cHE-LC12 cells, which appeared to be present in more than 30% of the area. Next, we analyzed the expression of the oncogene tumor protein 53 (TP53) in immortalized cell lines. The expression of TP53 of the four generated liver cancer cell lines are shown in Figure 3C,D. The four liver cancer cell lines expressed TP53 WT protein by exerting different levels of quantity, as shown in Figure 3D. The highest TP53 protein expression level was detectable in cHIB-LC4 cells compared to other cell lines. Nevertheless, TP53 expression levels from three out of four HCC cell lines were comparable to the expression level of immortalized HCC cell lines. The lowest expression level was detected for HepG2H1.3 cells.

### 3.5. Cross-Validation of Gene and Protein Expression Pattern in Patient-Derived Cells and Tissue Reveals Integrity of Generated HCC Cell Lines

After IF-based analysis and the characterization of the four newly generated HCC cell lines, we performed cross-validation by gene expression analysis of prominent expressed markers, like HNF4A, ALB, AFP, EGFR, CD44, and CK19, compared to patient-derived isolated cell fractions. As shown in Figure 4A, the expression pattern of analyzed HCC cell lines reflected the gene expression of parental cells derived from patient tissues, apart from the generated HCC cell line cHE-LC12. Here, generated HCC cells were negative for AFP mRNA production, while parental cells freshly isolated from the donor appeared to be positive. This finding was in line with the detection of the serum AFP level of patient 12 on the day of resection, as shown in Supplementary Figure S5C.

Next, we aimed to evaluate the repopulation of generated HCC cells in orthotopically transplanted mice. Therefore, we employed the cHB-LC11 cells after long-term cultivation. To identify cHB-LC11 cells in tissue slides derived from PDX mice, we used the continuously prominent tumor progression marker CD44, as well as the progenitor cell marker CK19, in combination with proliferation marker Ki67 and the pan MHC-class I Marker HLA-ABC. As shown in the representative Figure 4B (upper panel), vast areas of CD44-positive cells were detectable in the liver slides of transplanted mice after six months of proliferation and migration. At the same time, we could show, in Figure 4B (lower panel), a prominent occurrence of human Ki67-positive CD44-positive cells, indicating the proliferation capacity of those cells in the liver of immunosuppressed mice.

Human CK19-positive cells appeared in almost all transplanted mice by forming equal structures in the liver of the mice, as shown in the representative Figure 4C. The bile-duct-like structures of human CK19-positive cells appeared to be human HLA-ABC-positive in the basal region. In the interstitial areas, HLA-ABC-positive/CK19-negative cells were detectable. To evaluate the distribution of CD44- and CK19-positive HCC cells in the parental tissue, we employed the initial collected and paraffin-embedded tumor regions received from patient 11, as shown in Figure 4D,E. Here, we found comparable structures of CK19-positive cells (D) and high numbers of CD44-positive HCC cells (E).

In sum, we could follow up on the occurrence of CD44-positive and CK19-positive cells in parental tissue, in isolated patient-derived HCC cells, in cultured newly generated HCC cells and orthotopically transplanted mouse liver tissue, regarding the protein expression. Additionally, we could confirm the gene expression level of newly generated HCC cell lines compared to the four parental isolated cells.

### 3.6. Generated HCC Cell Lines Showed High Potential for Repeated Orthotopic Transplantation by Forming Discrete Sphere Colonies and Strong Cell–Cell Interactions

**Figure 5:**
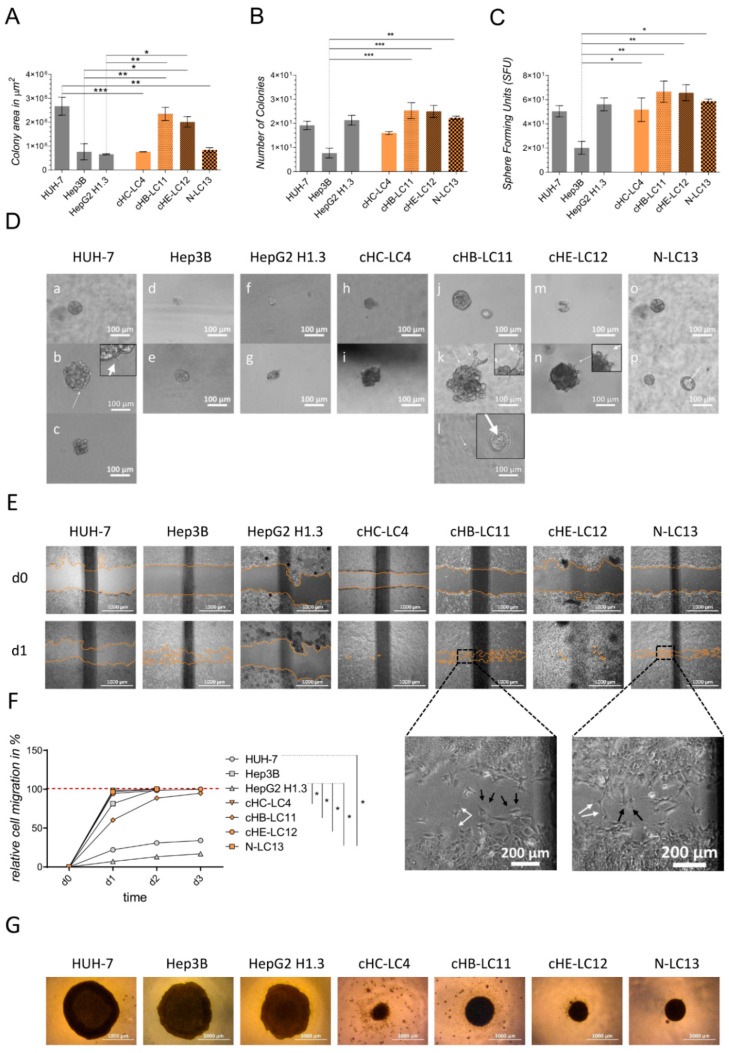
The migration and formation characteristics of freshly generated HCC cell lines compared to established ones. (**A**–**C**) Cell proliferation of indicated tumor cell lines based on their ability to form colonies in a 3D model in Matrigel. (**A**) Colony area in μm2 of indicated cell lines. (**B**) Counted number of colonies. (**C**) Sphere-forming units (SFU) by calculating (number of colonies/number of cells seeded) ×100% of each cell line. (**D**) Morphological changes are depicted through transmitted light microscope at a magnification of 10×, after cell growth for 11 days. Cell-line-specific changes in morphology like round and sleek (**a**,**d**,**f**,**h**,**j**,**o**) bubbly and textured (**b**,**c**,**e**,**g**,**i**,**k**,**l**,**n**), and hollow (white arrow, (**l**,**p**,**m**)) appearances are shown. In addition, invasive structures (white arrows (**b**,**k**,**n**)) are shown. (**E**) Scratch assay analysis to compare cell migration of indicated cell lines. Orange lines highlight the gap contour of the scratch. Closure of the cell-free area corresponds to relative cell migration in % over a period of up to 3 days. Close-ups of migrating cells of cHB-LC11 and N-LC13 depict types of cell migration like amoebic (black arrow, (**E**)) and polarity of a cell while migrating, consisting of a leading and trailing edge (white arrow, (**E**)). (**F**) Representative transmitted light microscopic images during scratch assay, day 0 and day 1. (**G**) Representative photographs of colonies under non-adherent conditions using PolyHEMA-coated plates. All statistical differences were characterized by plotting the p-value as follows: * *p* < 0.05; ** *p* ≤ 0.01 and *** *p* ≤ 0.001.

Generated liver cancer cell lines (cHIB-LC4, cHB-LC11, cHE-LC12, and N-LC13) and immortalized cell lines (HUH-7, Hep3B, and HepG2H1.3) were used to perform migration and colony-forming assays. The aim was to analyze their ability to form distinct tumors in vivo and in vitro. Therefore, the cell lines were cultured in a 96-well plate in a serum-starved medium Matrigel mixture and on PolyHema-coated 96-well plates with the appropriate culture medium. All cell lines were able to form spheres in an adhesion-independent manner. Representative images of liver cancer spheres on PolyHema are shown in Figure 5G and in Matrigel in Figure 5D. Both colony-forming methods showed different morphological structures of colonies. Aggregates of multiple cells on PolyHema presented large, flat, and loosely packed colonies (HUH-7, Hep3B, and HepG2H1.3), as well as smaller densely packed cell aggregates (cHIB-LC4, cHB-LC11, cHE-LC12, and N-LC13) as shown in Figure 5G. On the other hand, the clonogenic assay (Figure 5A–D) was based on the ability of liver cancer cell lines to form colonies through the proliferation of a single progenitor cell. By counting the number of colonies, we could assess the efficiency of colony formation and compare the growth characteristics of different cell lines. Proliferation was depicted as a colony area in µm^2^. We observed a significantly higher proliferation of re-cultivated cells after seeding 38 cells per well, at least in cHB-LC11 and cHE-LC12 cells, compared with Hep3B and HepG2H1.3 cells. The clonogenic survival of spheres, shown by the colony number and sphere-forming units (SFUs), uncovered significant differences between established cell line Hep3B and ex vivo liver cancer cell lines (cHIB-LC4 (SFU), cHB-LC11, cHE-LC12m and N-LC13 (colony number)). At the same time, cHB-LC11, cHE-LC12, and N-LC13 cells exhibited a higher tendency of colony-forming capacity in sphere number and SFU than reference cell lines. Furthermore, grown spheres revealed different growth patterns, as shown in Figure 5D. All cell lines appeared to be able to form compact colonies in Matrigel medium embedding. Round and smooth-shaped spheres were found in nearly all cell lines and seemed to appear in early stages and are cell-line-dependent in advanced stages of sphere formation, as investigated in all liver cancer cell lines. Bubble-like structures were presented by HUH-7, HepG2H1.3, cHB-LC11, cHIB-LC4, and cHE-LC12 cells (Figure 5(Db,Dc,Df,Dg,Dk,Dn)). Another cell-line-specific feature was identified by presenting hollow core structures in cHB-LC11 and N-LC13 cells (white arrows, Figure 5(Dl,Dp)). Furthermore, HUH-7, cHB-LC11, and cHE-LC12 cells formed invasive structures protruding into the Matrigel, even if only occasionally (white arrows, Figure 5(Db,Dk,Dn)). The migration ability of cells was investigated by performing a wound-healing assay, demonstrating a significantly enhanced motility of indicated in vitro liver cancer cell lines compared to HUH-7 and HepG2H1.3 cells (Figure 5F), which barely moved towards the gap. Progressing cell migration was illustrated by depicting wound healing at day 0 and day 1 (Figure 5E) and cells from different cell lines showed various migratory behaviour. Cells migrated individually, as indicated in Hep3B, cHE-LC12, and N-LC13 cells. Here, the typical leading edge and trailing edge of migrating cells (white arrows, Figure 5E) could be observed, implying loose cell–cell contacts and cell–matrix interactions. A collective movement in layers was also evident in HepG2H1.3, HUH-7, cHIB-LC4, and cHB-LC11 cells, which indicated tighter cell–cell interactions. Additionally, amoebic cell migration (white arrows, Figure 5E) could be seen occasionally in Hep3B, cHB-LC11, chE-LC12, and N-LC13 cells, demonstrating weaker cell–matrix connections.

In these experiments, the immortalized cell line HUH-7 cells exhibited the highest expansion and proliferation rate. In the case of liver cancer cell lines, specifically, cHB-LC11 and cHE-LC12 cells exhibited proliferation characteristics comparable to those of immortalized HCC cells but with the additional observation of denser cell clusters. These findings suggest potential differences in their growth and interaction dynamics within the tumor microenvironment. The liver cancer cell lines cHIB-LC4 and N-LC13 again showed comparable proliferation rates to Hep3B and HepG2H1.3 cells but also appeared in a denser cell cluster during the formation assay. In colony formation and colonial survival, all liver cancer cell lines were superior or equally potent compared to the immortalized target lines.

The differences in cell–cell contact formation led to the hypothesis that differences in the migration of the cell lines could be expected. Here, we found that all liver cancer cell lines ensured the complete overgrowth of the gap within a few days, like the Hep3B cells, whereas HUH-7 and HepG2H1.3 cells did not establish the contact in the scratch area and, thus, did not overgrow the gap at the same time. Embedding single cells in Matrigel also allowed us to investigate the cell proliferation rate. Immortalized cells, mostly Hep3B and HepG2H1.3 cells, showed the formation of only small colonies without the features of progressive proliferation like the infiltrative growth of spheres into the surrounding gel. The invasive and advanced growth of tumor cells in Matrigel may have been expected based on in vivo investigations of the massive tumor progression of immortalized HCC cell lines.

On the contrary, generated liver cancer cell lines demonstrated a higher proliferative capacity in vitro compared to the immortalized cell lines, with some showing a significant increase. As mentioned earlier, this was evident through their invasive growth and the formation of larger spheres. However, it is essential to note that liver cancer cell lines did not exhibit excessively progressive growth in vivo as shown in Supplementary Figure S4.

Nevertheless, there are similarities in the distribution of liver cancer cells in vivo with sphere formation from the cell aggregation of multiple cells in an anchorage-free environment on PolyHema. Here, the immortalized cell lines showed a planar and extensive growth in contrast to the conical spheres of freshly generated HCC cell lines. This characteristic of the established HCC lines is particularly relevant for in vivo studies, as the treatment of the highly tumorigenic and immortalized HCC cell lines does not reflect clinical reality.

### 3.7. Application of Cancer Treatments Results in Predictable Responses for Generated Liver Cancer Cell Lines

**Figure 6:**
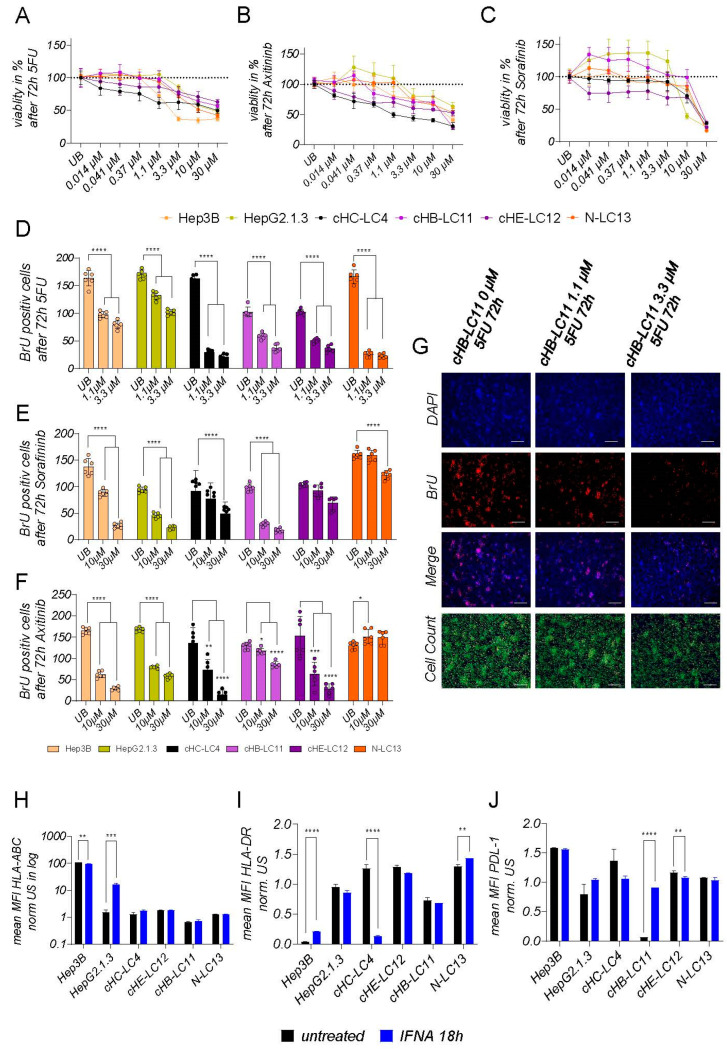
Reactivity under general treatment conditions of freshly generated HCC cell lines vs. immortalized cell lines. (**A**–**C**) MTT analysis of the toxicity of 5FU (**A**), Axitinib (**B**), and Sorafenib (**C**) after 72 h of treatment, ranking from untreated to 30 μM conditions. Viability of newly established HCC cell lines derived from mice after six months of transplantation compared to HepG2H1.3 and Hep3B cells and plotted in %. Plotted lines represent mean ± SD of 6 biological replicates per concentration. (**D**–**G**) BrU-based analysis of newly synthesized DNA after treatment with 5FU (**D**,**G**), Sorafinin (**E**), or Axitinib (**F**). The acquisition was performed using the macro cell count analysis from Keyence, as shown in (**G**). Here, counted results (below) were plotted against the single (DAPI, BrU) and merged staining. DAPI- and BrU-positive cells were counted automatically from 6 merge pictures per cell line and treatment, using the same macro count conditions along the cell lines. In (**E**,**F**), BrU-positive cells were counted after 72 h of incubation under indicated treatment options and were plotted against effective or highest conditions. The plotted bars represent mean ± SD of 6 biological replicates per concentration. In (**H**–**J**), flow-cytometer-based acquisition of surface marker HLA-ABC, HLA-DR, and PDL-1 from untreated or interferon-alpha-treated cells are blotted. Analyzed datasets represent the mean ± SD calculated from 3 technical replicates of acquired MFIs using antibodies against indicated surface marker normalized against unstained controls for background elimination. All statistical differences were characterized by plotting the *p*-value as follows: * *p* < 0.05; ** *p* ≤ 0.01 and *** *p* ≤ 0.001, **** *p* ≤ 0.0001.

As HCCs in patients display a highly heterogeneous cancer cell biology, the response to a particular treatment application can vary from non-responders to responders. The efficacy of anti-neoplastic drugs depends both on the tumor burden and its somatic variations. To analyze the response character of generated liver cancer cell lines and their capability to be used for drug screenings in vitro, we applied classical chemotherapeutic drug 5-FU, the tyrosine-kinase inhibitor Sorafinib, the protein-kinase inhibitor Axitinib, and interferon-alpha to these the four generated liver cancer cells, compared to immortalized cell lines Hep3B and HepG2H1.3. We analyzed the reduction in cell viability and metabolic changes (Figure 6A–C), and the ability to reduce the new synthesis of DNA (Figure 6D–G) for these drugs. As shown in Figure 6A, cell viability was decreased in all treated cell lines after 5-FU treatment, regardless of the entities, in high concentrations. At a clinical concentration of 1.1 µM 5-FU, cells of the generated liver cancer cell lines showed differences in response. Here, cHIB-LC4 and cHE-LC12 cells displayed a continuous reduction in cell viability starting at the lowest concentrations, in contrast to cHB-LC11 and N-LC13 cells, which are not responding at clinical concentrations. Both generated liver cancer cell lines showed slightly higher viability levels when applying lower concentrations of 5-FU. These findings aligned with the response of HepG2H1.3 cells after 72 h. With the expectance of Hep3B cells, all analyzed cell lines showed a continuously higher effect in dependence on higher concentrations.

CHE-LC12 cells showed an exclusive response for multikinase inhibitor sorafenib, as shown in Figure 6C. Interestingly, in cHB-LC11 and HepG2H1.3, a low-concentration treatment induced enhanced cell viability and metabolism. In HepG2H1.3 cells, a 10 µM concentration significantly reduces cell viability, while cHB-LC11 shows a therapeutic effect only when using very high unphysiological concentrations of 30 µM. CHIB-LC4, N-LC13, and Hep3B cells show slide toxicity when applying concentrations from 3.3 µM. With higher concentrations, the toxicity of sorafenib was increased.

The application of downstream tyrosine-kinase inhibitor Axitinib affected cell viability very heterogeneously. Here, cHIB-LC4 and cHE-LC12 cells were, similarly to the case after 5-FU treatment, continuously reduced in cell viability with increasing concentrations. HepG2H1.3 and cHB-LC11 and N-LC13 cells show increased cell viability when applying low concentrations of Axitinib. The induction of toxicity was detectable starting at 0.37 µM for cHB-LC11 cells, whereby a dose of 1.1 µM or even 3.3 µM was effective in HepG2H1.3, N-LC13, or Hep3B cells, respectively. Cell viability reduction was detectable for all cell lines when reaching a dose of 3.3 µM Axitinib, pointing out that HCC cells respond better to Axitinib than to Sorafinib. The interference with DNA synthesis is shown in Figure 6D–G. After application of 5-FU, interference was most prominent, as shown in Figure 6D,G. Multikinase inhibitor Sorafinib application resulted in an effective reduction of DNA synthesis in cHB-LC11, HepG2H1.3, and Hep3B cells.

In contrast, in cHIB-LC4, cHE-LC12, and N-LC13 cells, only slight effects of DNA synthesis interference were observed. Determining the impact of the tyrosine-kinase inhibitor Axitinib for N-LC13 cells, there was no effective interference with DNA synthesis detectable. A mild inference effect was detectable in cHB-LC11 and HepG2H1.3 cells while the treatment of Hep3B, cHE-LC12, and cHIB-LC4 cells with high concentrations of Axitinib led to a continuous reduction of DNA synthesis. We could show that liver cancer cell lines exert equal or even stronger cytotoxic reactions on applying drugs compared to immortalized cell lines. These findings point out that all four cell lines can be used for drug screening, dosage-finding experiments, and combination therapy in vitro.

The application of interferons can significantly suppress tumor growth in HCC but, on the other hand, relatively increase the number of circulating tumor cells [26]. Interferon application can alter the presentation of surface proteins on HCC cells and lead to anti-viral effects by inducing apoptosis [27]. Here, we examined the general effect of IFN-alpha on our generated liver cancer cell lines. As shown in Figure 6H–J, the application of interferon-alpha results in almost no elevation of HLA-ABC in tested cell lines for the tested timepoint, except for HepG2H1.3 cells, which were genetically modified to produce viral HBV particles. Only a slide trend in the increased presentation was measurable in cHB-LC11 and cHIB-LC4 cells, which are viral-induced liver cancer cell lines. The presentation of HLA-DR was altered in all cell lines without a clear, understandable pattern. Only in cHIB-LC4 cells was a remarkable reduction of HLA-DR expression detectable after 18 h of IFN-alpha treatment. After 18 h of IFN-alpha treatment, the presentation of PDL-1 was slightly decreased in cHC-4-LC, cHE-LC12, and N-LC13 cells, and induced in HBV-derived cHB-LC11 cells, and, therefore, reacted against the inflammatory effect of interferon-alpha.

Here, we demonstrate the response of generated liver cancer cell lines for treatment options that interfere with different cellular pathways and functions. We could show that generated liver cancer cells respond to varying levels of different clinically used treatments in vitro, which reflected the heterogeneity of HCC in patients.

## 4. Discussion

Hepatocellular carcinoma is a highly prevalent malignancy and is a common cause of cancer-related mortality. It is estimated that, by 2025, more than 1 million patients will develop liver cancer annually. The hepatitis virus infection is the leading cause in approximately 50% of HCC cases, whereas, currently, non-alcohol-related steatohepatitis (NASH) is rapidly becoming a growing etiological concern. Most patients with a developed HCC are diagnosed when reaching the advanced disease stage and are, therefore, lacking effective treatment options. Consequently, novel molecular biomarkers are needed to predict tumor therapy and evaluate prognosis.

On the other hand, we need relevant pre-clinical models to give novel drug candidates access to clinical studies and approvals. In liver cancer research, human-derived immortalized HCC cell lines like Hep3B and HUH-7 have a long history in pre-clinical testing strategies and played, therefore, a remarkable role by leading to a better understanding of tumor biology and supporting the development of new drugs [13,28,29]. These cell lines’ advantages are their easy handling, robustness [30], and almost gapless understanding of their genomic, transcriptomic, and proteomic landscape. However, due to their genetic alterations, altered growth rates, and adapted metabolic pathways, immortalized cell lines deviate significantly from the characteristics of the patient-derived cells [31].

As a result, these cell lines lost the ability to accurately mimic the clinical context, which leads to a reduced capacity for providing comprehensive insights into drug efficacy or the underlying mechanisms. Here, individual liver cancer material from patients can provide more accurate and substantiated results regarding drug efficacy [32]. The material can be used in fresh culture or xenotransplantation in immunosuppressed mice to preserve the patient’s imprint. Fresh tumor tissue culture can be maintained over a short period in vitro. However, it cannot be used for repeatable comparative studies of different therapeutic approaches [11], as in vivo xenograft models cannot be used for pre-screenings due to cost and ethical reasons. The only reasonable and affordable way to solve this problem is to generate a combined workflow for in vivo and in vitro cultivation of the primary liver cancer cells. In this regard, we established a complete isolation and cultivating workflow for patient-derived HCCs to facilitate a long-term cultivation of viable individual liver cancer cells and associated cells.

A total of 15 resections were processed for this study and used for the advanced protocol. Our study revealed that 64% of the isolated samples exhibited the capability for short-term 2D cultivation, which displayed a higher success rate than others [15,33,34]. Due to their underlying clinical parameters, patients 4, 11, 12, and 13 have been chosen to generate patient-derived liver cancer cell lines. Therefore, they were employed for orthotopic transplantation into the liver of immune-deficient NSG mice. Remarkably, all four transplanted samples were suitable for repopulation of the mouse liver and were, moreover, suitable for long-term in vitro cultivation. By conducting ex vivo cultivation, we observed the presence of cancer-associated fibroblasts (CAFs) and cancer stem cells (CSCs). As supported by previous studies, CAFs, or tumor-associated fibroblasts, exhibit high versatility within the tumor environment [35]. Their presence and interactions are believed to contribute significantly to the overall behaviour and characteristics of the cultured cells [36]. In line with others, we observed that CAFs play a critical role in the formation and adaptation processes within 2D culture [37]. After the initial formation of CAF assemblies, the process of forming individual colonies, including liver cancer cells, takes place, finally allowing the development of a monolayer. This transition from cell assemblies to a monolayer structure represents a crucial stage in the cultivation process, as it establishes a more organized and cohesive cellular arrangement. Besides their general ability of cell cluster formation and migration, the generated liver cancer cell lines exhibited individual highly heterogeneous characteristics.

The comparison of CSC marker presentation in the isolated cell fractions from the patient and mouse material reveals both the induction of diversity and the elevated percentage of CSC-specific markers in three out of four samples after repopulation of the mouse liver. In sample number four (cHE-LC12), at least the carcinogenesis-promoting factor CD13 was upregulated. Considering that CSCs are providing the ability for the self-renewal and differentiation of the liver cancer cells, as well as the tumorigenesis, the repopulation and proliferation in the physiological environment of the mouse liver increases the potential to grow in vitro for all four samples. These results point out that liver cancer cells must be at least positive for CD13 and for CD73 to be suitable for in vitro long-term cultivation. The expression of CD73, which is associated with increased aggressiveness and promotion towards metastasis formation [38], was found to be most prominent in all HCC cell lines and even more pronounced in established HCC cell lines. These findings are consistent with the highly tumorigenic progression observed in xenograft mice. The four liver cancer cell lines exert a different allocation of CSC-specific surface antigens, pointing to their high HCC heterogeneity compared to HepG2.1.3 and Hep3B cells. The high heterogeneity was also obtained by performing the cellular staining of different hepatocyte and cancer-cell-specific proteins. All four cancer cell lines were human-specific and showed different cell types. CHB-LC11, cHIB-LC4, and cHE-LC12 are CK19-positive HCCs and are, therefore, interesting since this kind of liver cancer was shown to be chemo-resistance. Liver cancer cell lines displayed a dual phenotype, with characteristics resembling both hepatocytes and cholangiocytes and variations depending on the specific liver cancer cell line. Notably, the NASH-derived N-LC13 line exhibited a predominantly cholangiocyte-like character, while the viral-induced cell lines cHB-LC11, cHIB-LC4, and cHE-LC12 displayed a predominantly hepatocyte-like phenotype. These observations indicate heterogeneity in the differentiation state or lineage commitment of the HCC cell lines, potentially reflecting the diverse cellular subpopulations within the parental tumor. In addition, we identified immunosuppressive characteristics in certain cell lines based on the presence of programmed cell death ligand 1 (PD-L1), albeit in low frequency. Specifically, the virus-induced HCC cell lines cHB-LC11, cHE-LC12, cHIB-LC4, and HepG2H1.3 presented PD-L1 expression, suggesting the potential efficacy of PDL-1 inhibitors. The presence of CD68-positive cells within the liver cancer cell lines was particularly intriguing, as CD68 is known as a marker for cancer-associated macrophages (CAMs) but is also present in other liver-associated cell types, like hepatocytes. Further analysis needs to be performed to better understand the role and potential of the CD68+ population in the generated cell lines. Cancer-associated macrophages (CAMs) have diverse roles in the tumor microenvironment, including immune response modulation, tumor promotion, and angiogenesis [39]. Thus, it can be estimated that CD68-positive cells can be supportive of combined immune therapies, as they were positively related to the infiltration of immune cells in many tumor types [40].

Comparing the generated liver cancer cell lines to immortalized cell lines, the aggregation and colony-forming assays revealed that liver cancer cell lines tend to form more dense cell aggregates and stronger cell–cell interactions. In contrast, the observed immortalized cell lines require less cell–cell interaction for aggregation. The highest and, therefore, unphysiological expansion was obtained using HUH-7 cells in vitro and in vivo. These results show that HUH-7 cells cannot reflect the clinical stage of tumor progression and, therefore, cannot be used for the xenograft experiments in mice. The cell line Hep3B was not aggressive to that high intent but appeared to form extensive, highly necrotic areas in the liver of NSG mice. In contrast, generated liver cancer cell lines form dense clusters in the provided matrices. Therefore, they can mimic the clinical level of progression, as confirmed by orthotopic transplantation experiments.

Moreover, by cross-validation of tumoral general highly expressed marker CD44 and CK19 in patient- and mouse-derived tissue samples, we could confirm the integrity of newly generated HCC cell lines. The generated HCC cells showed solid migration accomplished by proliferation and were able to form similar structures of CK19-positive cells in the liver of mice compared to the parental patient-derived tissues.

The general ability of generated liver cancer cell lines regarding their treatment-related response points out that all four lines are suitable to maintain pre-screening experiments for therapy options in viral-induced and NASH-related HCCs. They reveal differences in the response and non-response to different drugs and, therefore, can be used also for combination therapy investigations. Moreover, as they exhibit less aggressive but dense formations in the mouse liver environment, they can be used at the same time to evaluate therapy options in vivo in xenografted NSG mice rather than immortalized cell lines.

A suitable prediction of a combined effective therapy would be the combination of a PDL-1 inhibitor with IFN alpha in cHB-LC11 cells. In the cHB-LC11 xenograft model, engineered effector T-cells with integrated PDL-1 inhibitory signals can be used to facilitate a solid anti-tumor effect.

## 5. Conclusions

In conclusion, we demonstrated in this study an approach to generating individual liver cancer cell lines that exhibit a heterogeneous presentation of cancer stem cells, primary cancer cell characteristics, and the potential and ability to form tumors in a clinically relevant manner in vitro and in vivo, and can be maintained for treatment experiments, in vitro and in vivo. Therefore, the impact of the study lies in the possibility of extensively studying individual HCC cell lines generated from patient material in vitro before the same individual cells can be used for an in vivo evaluation of the findings. With regard to immune therapies, which will not be standalone therapies, the investigation of combinations with common or new drugs will be the future [41,42,43,44]. Therefore, both the in vitro and the in vivo examination are possibly conducted in the same cells.

## Data Availability

Data are contained within the article or its Supplementary Materials. Data, analytic methods, and study materials can be made available to other researchers by requesting their usage for other studies by the corresponding author.

## Supplementary Materials

The following supporting information can be downloaded at: https://www.mdpi.com/article/10.3390/cells13010082/s1, Figure S1: Representative flow cytometry gating strategy for identification of CSC marker epitopes. Figure S2: Representative analysis output data from macro cell counts used to quantify BrU positive pHCC-cells. Figure S3: Gene expression level of tissue sample from patients. Figure S4: Orthotopic transplantation of stable luciferase transduced HCC cell line HUH-7, Hep3B vs. pHCC cell lines. Figure S5: Immunohistochemical staining of HepG2H1.3-and pHCC-cells against murine antigens. Table S1: Table of Antibodies used for IHC and IF staining. Table S2: List of TaqMan Assays.

## Abbreviations

5-FU	5-Flour-Uracil
CAF	cancer-associated fibroblasts
CAM	cancer-associated macrophages
CSC	cancer stem cells
HBV	hepatitis B virus
HCC	hepatocellular carcinoma
HCV	hepatitis C virus
NAFLD	non-alcoholic fatty liver diseases
PBMC	peripheral blood mononuclear cells
PDX	patient-derived cells
pHCC	primary-derived HCC
SFU	sphere-forming units
TIL	tumor-infiltrating lymphocytes
TP53	tumor suppressor 53
HEV	hepatitis E virus
IFN	interferon
