# Supplementary material for: Generating Patient-Derived HCC Cell Lines Suitable for Predictive In Vitro and In Vivo Drug Screening by Orthotopic Transplantation
Source: Cells. 2023 Dec 30;13(1):82. doi: 10.3390/cells13010082 (PMC10778205; doi:10.3390/cells13010082)
Supplement: Supplementary file 1 [file cells-13-00082-s001.zip › cells-2759063-supplementary.pdf]

Supplementary Figure S1

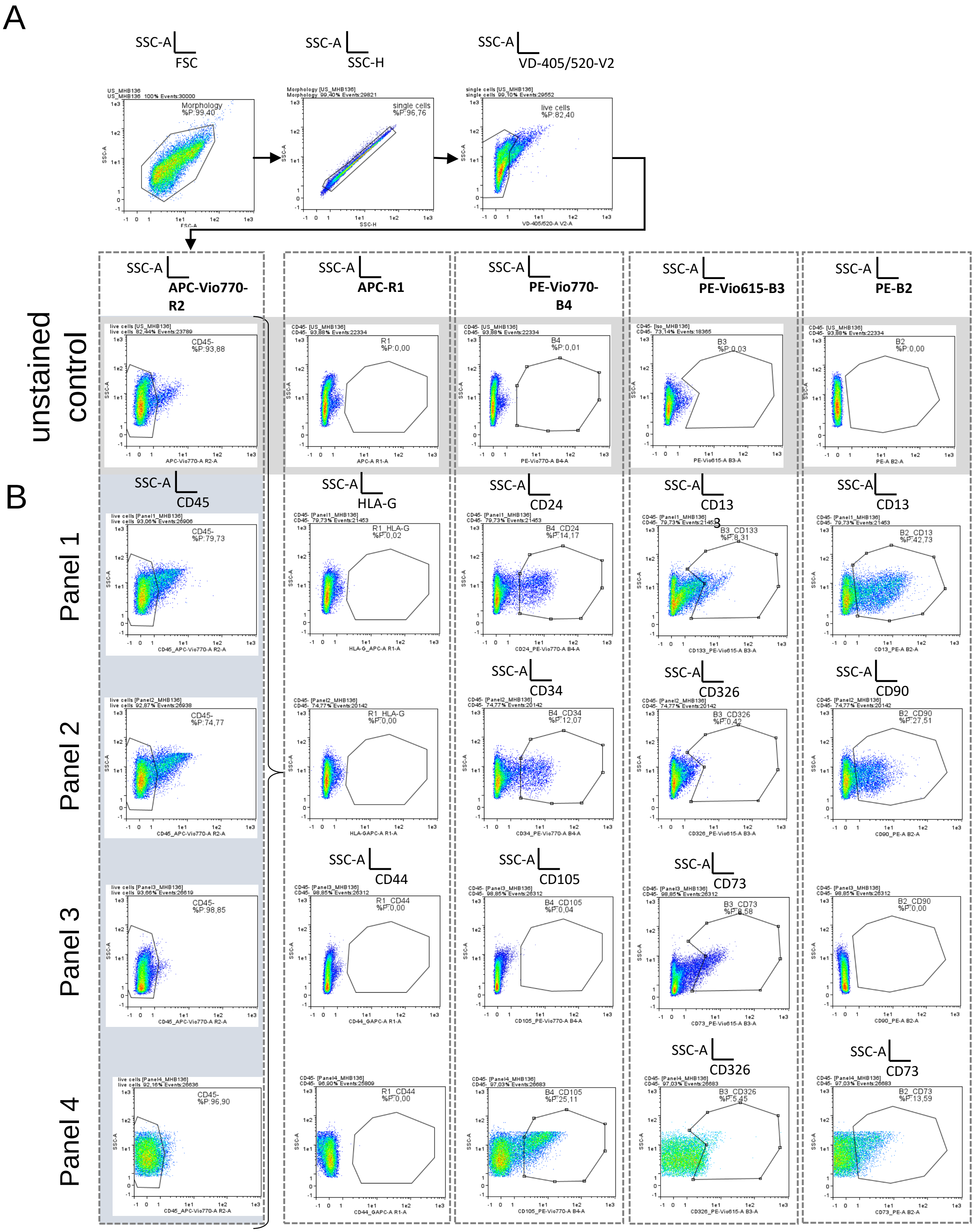

Supplementary Figure S1

Representative flow cytometry gating strategy for identification of CSC marker epitopes on the surface of pHCC-cells. (**A**) Using a side scatter area (SSC-A) versus forward scatter (FSC) area the debris was excluded and cells of interest were included. Cells were gated on singularity by SSC-A versus SSC-Height (SSC-H) and further eliminated by excluding ViobilityDye (VD)-105/520 positive dead cells. Live cells feature the starting point for each CSC-panel's immune cell (CD45 positive) exclusion (blue background). Unstained pHCC-cells (grey background) of respective cell lines were used as negative controls to define fluorochrome-positive cells in the particular detection channel (APC-Vio770 represents channel R2, APC-R1, PE-Vio770-B4, PE-Vio615-B3 and PE-B2 versus SSC-A). (**B**) Each Antibody-Panel (1-4) represents an antibody-cocktail of five CSC-specific antibodies (Ab). Antibodies (Abs) in Panel 1 are specific for the epitopes HLA-G-R1, CD-24-B4, CD133-B3 and CD13-B2. Panel 2 includes Ab against HLA-G-R1, CD34-B4, CD326-B3 and CD90-B2. Panel 3’s Abs are specific for CD44-R1, CD105-B4, CD73-B3 and CD90-B2. Panel 4 compromises CD44-R1, CD105-B4, CD326-B3 and CD73-B2 specific Abs.

Supplementary Figure S2

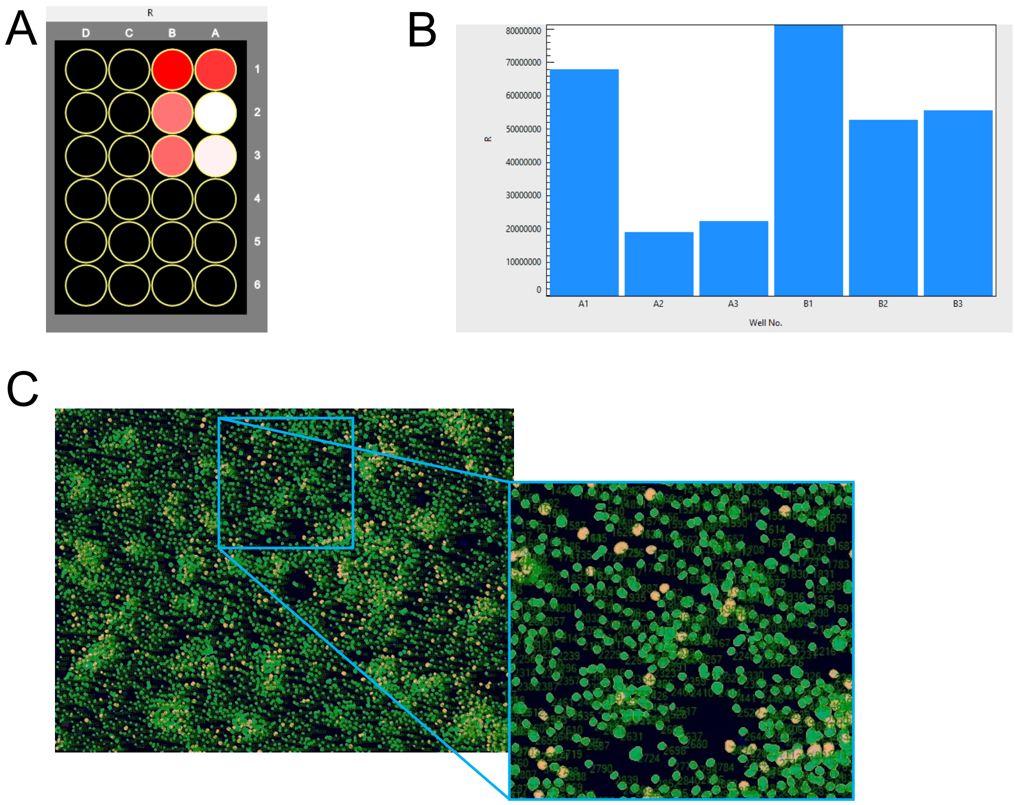

Supplementary Figure S2

Representative analysis output data from macro cell counts used to quantify BrU positive pHCC-cells.

(**A-C**) Output data generated by the Macro cell count program from Keyence. (**A**) Displays scanned plate and well position, which was counted. A1, B1 = untreated controls, A2, A3 represent pHCC-cells treated with 3,3µM 5-FU and B2, B3 present the pHCC-cells treated with 1,1µM 5-FU. The overall intensity displayed in A was plotted in (**B**) against counted wells; well IDs are mentioned at the x-axis. In every well, six positions were counted under the same conditions (position in well and extension). A representative merged and counting capture is displayed in (**C**), including numbers for the counting area = DAPI stained nuclei (green) and the target area stained in Alexa-555 BrU assembled new synthesized DNA (yellow).

Supplementary Figure S3

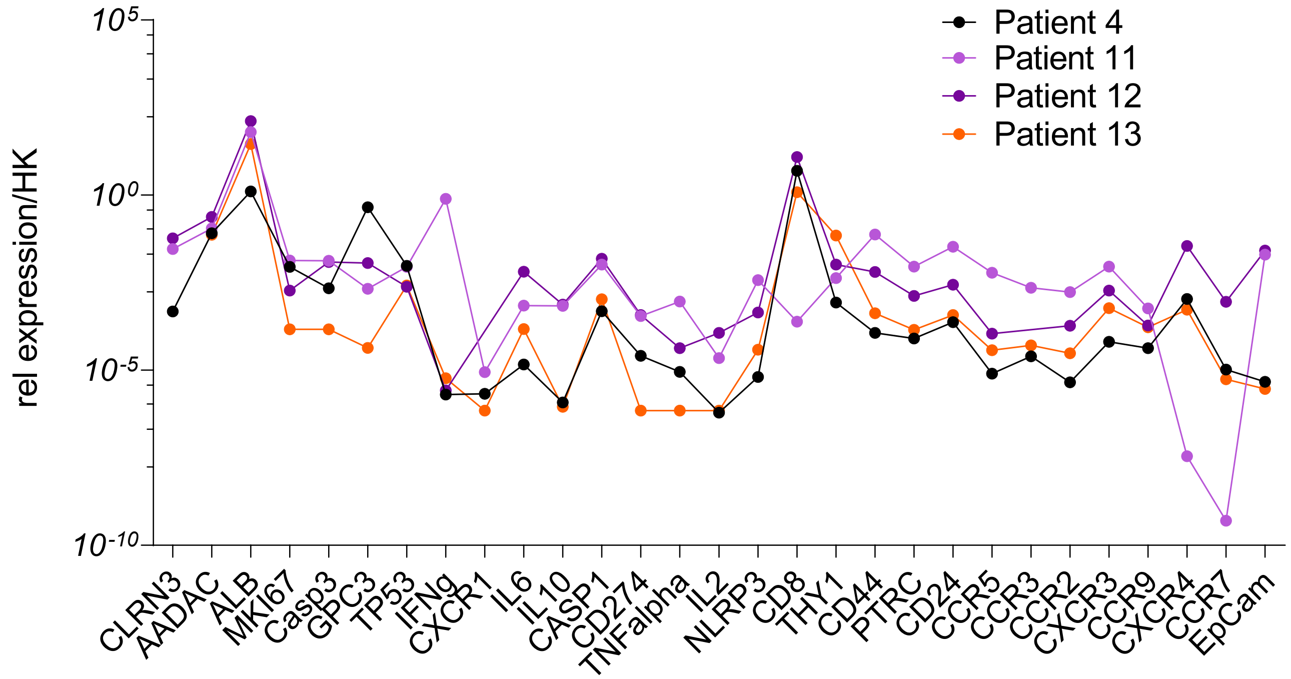

Supplementary Figure S3

Gene expression level of tissue sample from patients.

Determination of liver-specific and inflammatory markers at RNA level from tissues derived from patient material included in the study. RNA gene expression data were normalized against human-specific housekeeper genes GapDH and RPL30.

Supplementary Figure S4

A

Maus ID	cells transplanted	time tumor growth	Photons total flux (p/s)
106	HUH-7_Luc	3,14	2.6E+09
92	Hep3B_Luc	5,1	7.8E+08
247	cHB-LC11_Luc	15	7.30E+07
250	cHB-LC11_Luc	15	1.20E+07

B

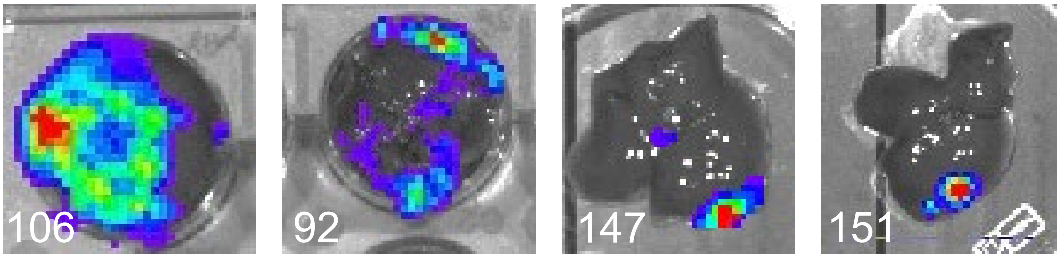

Supplementary Figure S4

Orthotopic transplantation of stable luciferase transduced HCC cell line HUH-7, Hep3B vs. pHCC cell lines.

(**A**) Table of transplanted mice included ID, cell type, time of tumor development and measurement of total bioluminescence *in-vivo* before scarification. (**B**) Overlay of photographs and bioluminescence measurement from mouse livers after scarification. Auto-modus detected bioluminescence; luciferin injection was performed 10min before detection.

Supplementary Figure S5

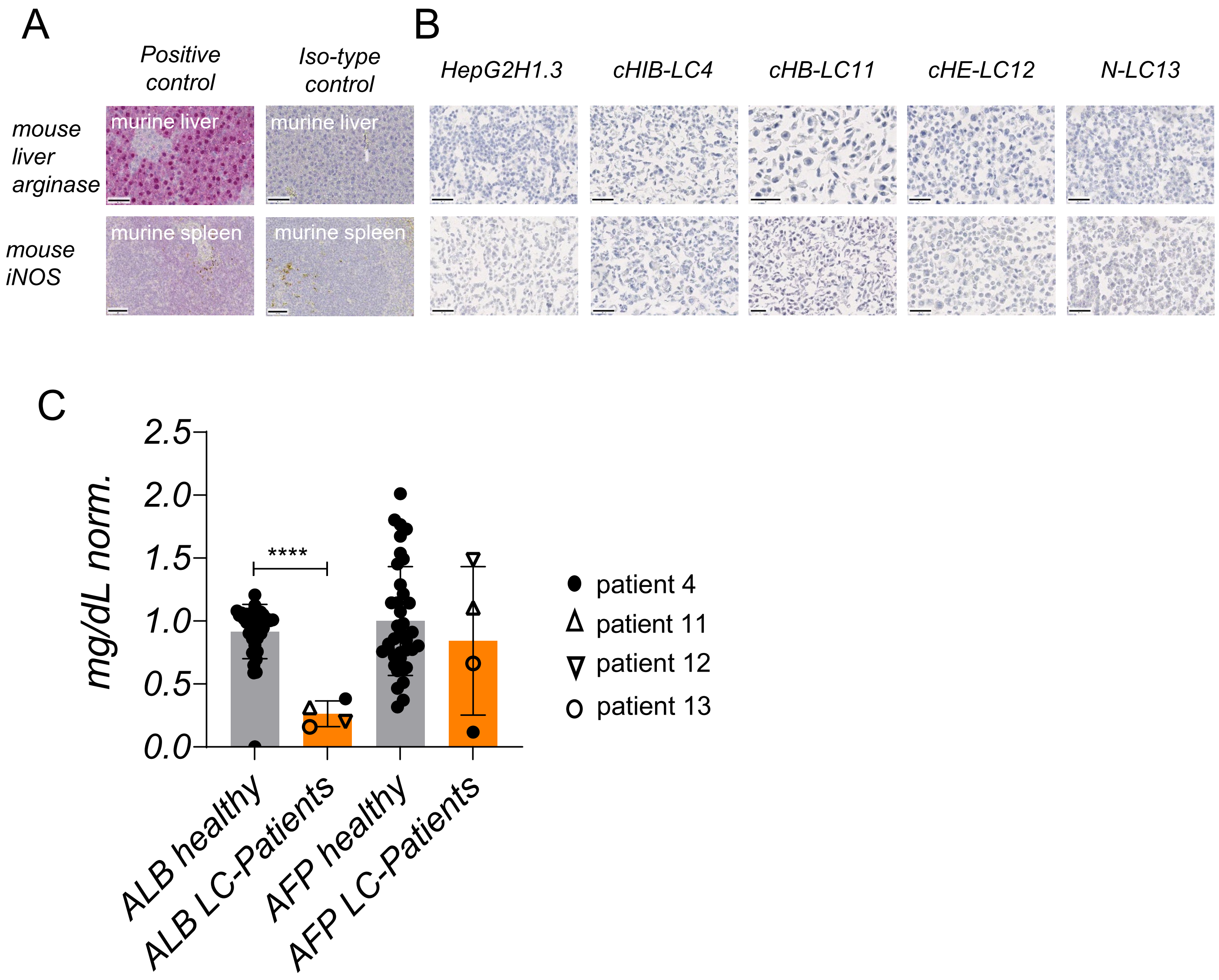

Supplementary Figure S5

Immunohistochemical staining of HepG2H1.3-and pHCC-cells against murine antigens. (**A**) Murine reference tissues expressing Liver Arginase (murine liver) and iNOS (murine spleen) were used for positive and negative isotype control staining. (**B**) Cell pellets of immortalized cell line HepG2H1.3 agar and pHCC cell lines embedded in the agar were stained against murine Liver Arginase and iNOS to verify no cross-contamination with murine cells in cell culture after pHCC cell isolation. (**C**) Determination of human Alpha-fetoprotein and human Albumin protein levels in the plasma samples obtained from whole blood samples (n=4) after PBMC isolation procedure. Plasma levels were plotted against a healthy donor (n= 36) cohort and normalized against background controls. Bar chart represents mean ± SD; ****p ≤ 0.0001 using a two-tailed unpaired t-test.

Supplementary Table S1: Table of Antibodies used for IHC and IF staining

Antigen	Producer	Target	Company
Vimentin	mouse monoclonal	human	Santa Cruz
CD31	mouse polyclonal	human, mouse, pig	Invitrogene
CK18	mouse monoclonal	human	ExBIO
PD-L1	rabbit polyclonal	human	GeneTex
CD68	mouse monoclonal	human	Dako
Calnexin	rabbit monoclonal	human	ThermoFischer
CD44	rabbit Polyclonal	human, mouse, rat	ThermoFischer
NTCP	rabbit monoclonal	human	ThermoFischer
HNF4	goat polyclonal	human	ThermoFischer
CD24	rat polyconal	human	ThermoFischer
Ki67	rat monoclonal	human	Origene
Actin	rabbit Polyclonal	human, mouse	Abcam
CK19	rabbit monoclonal	human	Avivasysbio
EpCam	mouse monoclonal	human	Origene
Desmin / CD33	mouse monoclonal	human	Dako Aglient
Alpha Anti Trypsin	mouse monoclonal	human	Biotrend
CD90	mouse monoclonal	human	BD Bioscinece
EGFR	mouse monoclonal	human	Thermo Fischer
CK7	mouse monoclonal	human	Dako Aglient
CK8	mouse monoclonal	human	santa cruz
AFP	mouse monoclonal	human	Thero Fischer
Caspase3	rabbit Polyclonal	human	Invitrogene
AADAC	rabbit Polyclonal	human	proteintech

Supplementary Tabel S2: List of TaqMan Probes

Probe	Assay-ID
GapDH	Hs99999905_m1
RPL30	Hs00265497_m1
ALB	Hs00609411_m1
AFP	Hs01040598_m1
CD44	Hs01075864_m1
HNF4A	Hs00230853_m1
EGFR	Hs01076090_m1
CK19	Hs00761767_s1
